# Piperlongumine Acts as an Immunosuppressant by Exerting Prooxidative Effects in Human T Cells Resulting in Diminished T_H_17 but Enhanced T_reg_ Differentiation

**DOI:** 10.3389/fimmu.2020.01172

**Published:** 2020-06-12

**Authors:** Jie Liang, Jacqueline D. Ziegler, Beate Jahraus, Christian Orlik, Renata Blatnik, Norbert Blank, Beate Niesler, Guido Wabnitz, Thomas Ruppert, Katrin Hübner, Emre Balta, Yvonne Samstag

**Affiliations:** ^1^Section Molecular Immunology, Institute of Immunology, Heidelberg University, Heidelberg, Germany; ^2^Mass Spectrometry Core Facility, Center for Molecular Biology (ZMBH), Heidelberg University, Heidelberg, Germany; ^3^Division of Rheumatology, Department of Internal Medicine V, Heidelberg University, Heidelberg, Germany; ^4^Department of Human Molecular Genetics, Heidelberg University, Heidelberg, Germany; ^5^nCounter Core Facility, Department of Human Molecular Genetics, Heidelberg University, Heidelberg, Germany

**Keywords:** piperlongumine, primary human T cells, reactive oxygen species, glutathione, T_H_17 cells, T_reg_ cells

## Abstract

Piperlongumine (PL), a natural small molecule derived from the *Piper longum* Linn plant, has received growing interest as a prooxidative drug with promising anticancer properties. Yet, the influence of PL on primary human T cells remained elusive. Knowledge of this is of crucial importance, however, since T cells in particular play a critical role in tumor control. Therefore, we investigated the effects of PL on the survival and function of primary human peripheral blood T cells (PBTs). While PL was not cytotoxic to PBTs, it interfered with several stages of T cell activation as it inhibited T cell/APC immune synapse formation, co-stimulation-induced upregulation of CD69 and CD25, T cell proliferation and the secretion of proinflammatory cytokines. PL-induced immune suppression was prevented in the presence of thiol-containing antioxidants. In line with this finding, PL increased the levels of intracellular reactive oxygen species and decreased glutathione in PBTs. Diminished intracellular glutathione was accompanied by a decrease in S-glutathionylation on actin suggesting a global alteration of the antioxidant response. Gene expression analysis demonstrated that T_H_17-related genes were predominantly inhibited by PL. Consistently, the polarization of primary human naïve CD4^+^ T cells into T_H_17 subsets was significantly diminished while differentiation into T_reg_ cells was substantially increased upon PL treatment. This opposed consequence for T_H_17 and T_reg_ cells was again abolished by thiol-containing antioxidants. Taken together, PL may act as a promising agent for therapeutic immunosuppression by exerting prooxidative effects in human T cells resulting in a diminished T_H_17 but enhanced T_reg_ cell differentiation.

## Introduction

PL is a compound that can be isolated from different piper species, especially from *Piper longum* Linn (*P. longum*, Indian long pepper). It has been traditionally used in Asia for the treatment of gastrointestinal complaints, respiratory diseases and infectious diseases, such as malaria (1). In recent years, PL has gained increasing interest as a potential drug against cancer. The anticancer effects were mainly shown by an inhibition of the proliferation of various human cancer cell lines under *in vitro* conditions and an increased cancer cell death in the presence of PL (2–5). Anticancer effects of PL have also been found *in vivo* in mouse xenograft tumor models (4, 6, 7). Based on that, several patents have been filed for the treatment of cancer using PL and PL analogs (8). Mechanistically, PL is a prooxidative compound that increases the amount of reactive oxygen species (ROS) in cancer cells, which is directly linked to PL-associated anticancer activities (9, 10). Further studies could identify several ROS-dependent signals, e.g., PI3K/AKT/mTOR (4) and NF-κB pathways (11–13), signal transducer and activator of transcription (STAT) 3 (7) and p38 (3, 14) as targets of PL in cancer cells. PL also interacts with biologically important small molecules, e.g., it is considered as a direct inhibitor of the thioredoxin reductase 1 in human gastric cancer cells, which leads to ROS accumulation and ROS-dependent cell death (15). Moreover, in cancer cells PL was reported to deplete the reduced glutathione (GSH) stores (16) and to inactivate other thiol-containing proteins involved in maintaining cellular redox homeostasis through thiol modification (5). Besides, PL induces endoplasmic reticulum stress (17) and inhibits the ubiquitin-proteasome system (18), which are also linked to increased ROS levels.

Despite intensive research on its anticancer properties, potential effects of PL on human immune cells, especially T cells, were disregarded in initial studies in this field. In tumor patients potent anti-tumor immune responses are extremely important, e.g., for successful immunotherapy. We, therefore, asked whether treatment with PL affected the function of human T cells. Earlier studies, in which the effects of PL in chronic inflammatory diseases were analyzed, provided first insights into the immunomodulatory properties of PL in the mouse system. *Xiao* et al. demonstrated, under *in vitro* conditions, that PL inhibits the LPS-induced maturation of mouse bone marrow-derived dendritic cells (DCs). This observation was confirmed by *in vivo* experiments showing decreased maturation of splenic DCs in mice with collagen-induced arthritis (CIA) (19). Furthermore, *Sun* et al. have shown in a mouse model of CIA that PL expanded myeloid-derived suppressor cells (MDSC) and reduced the arthritis score and histopathologic lesions (20). Another study reported that PL improved the symptoms of lupus nephritis in MRL-Fas (lpr) mice by decreasing the levels of proinflammatory cytokines and the frequency of T_H_17 cells while increasing the frequency of T_reg_ cells (21). In line with the shifted T_H_17/T_reg_ ratio, PL ameliorated MOG-induced experimental autoimmune encephalomyelitis (EAE) in mice due to dampened NF-κB signaling (22). PL also inhibited the activation and function of human fibroblast-like synoviocytes (FLS) that were derived from rheumatoid arthritis (RA) patients (20, 23). However, a potential direct influence of PL on human T cells has not been investigated. Given the crucial role of T cells in the immune system, it is, however, important to know whether and how PL affects T cell immunity in the human system in order to assess its potential clinical benefit.

The molecular mode of action of PL on various immune cell types is only partially known. In contrast to the situation in cancer cells, in splenic DCs PL diminished the intracellular ROS levels, thereby suppressing the maturation of DCs (19). Likewise, PL decreased TNFα-induced intracellular ROS production in RA FLS (23). Overall, a confusing picture emerged with regard to the effects of PL on the intracellular redox homeostasis in different cell types.

T cell functions can be strongly modulated by influencing the actin cytoskeleton via the redox balance (24). In addition, lowered ROS levels in T cells of RA patients were reported to promote their differentiation into IL-17 and IFNγ-producing inflammatory cells (25). Vice versa, a prooxidative microenvironment reduced the cytokine response of terminally differentiated T_H_1 and T_H_17 cells (26). Furthermore, *Fu* et al. reported that accumulation of ROS active misshapen/NIK-related kinase 1 (MINK1) limited the generation of T_H_17 cells. This phenotype was prevented in the presence of the antioxidant N-acetyl-cysteine (NAC) (27). Along the same line, inhibition of pyruvate dehydrogenase kinase (PDHK) specifically impaired T_H_17 cells, while sparing T_H_1 and promoting T_reg_ cells. Again, this was mediated in part through ROS since NAC treatment restored T_H_17 cell generation (28). Based on the reported redox regulation by PL, our goal was to investigate whether PL serves as a novel means to modulate the redox balance and thus the function of primary human T cells.

In the present study, we found that PL was not toxic to primary human T cells, as opposed to the malignant T leukemia line Jurkat. However, PL inhibited the activation and proliferation of primary human T cells after costimulation through CD3 and CD28. In particular, the development of proinflammatory T_H_17 cells was significantly diminished, while anti-inflammatory T_reg_ cells were upregulated. We were able to attribute these results to the prooxidative activity of PL in primary human T cells, because PL increased intracellular ROS levels and decreased GSH levels, and the PL-mediated immunosuppressive effects were weakened by thiol-containing antioxidants. Furthermore, we identified redox-regulated targets of PL, e.g., hypoxia-inducible factor (HIF)-1α.

Together, our data demonstrate that PL acts as an immunomodulating agent for primary human T cells. By altering the redox balance toward a prooxidative milieu PL shifts T cell immunity toward an immunosuppressive phenotype. Thus, PL may represent a novel option to control autoimmune disorders.

## Materials and Methods

### Materials

Piperlongumine (#SML0221), DMSO, NAC, Paraformaldehyde (PFA), phorbol 12-myristate 13-acetate (PMA), Ionomycin and Brefeldin A were purchased from Sigma-Aldrich (St. Louis, USA). GSH, 5-(and-6) chloromethyl-2′, 7′-dichlorodihydro-fluorescein diacetate, acetyl ester (CM-H_2_DCFDA), RPMI 1640 and ThiolTracker^TM^ Violet were purchased from Thermo Fisher Scientific. Carboxyfluorescein diacetate succinimidyl ester (CFSE) was bought from Invitrogen (Eugene, USA) and fetal bovine serum (FBS) from PAN-Biotech (Aidenbach, Germany). nCounter® GX Human Immunology v2 panel was purchased from NanoString Technologies and Direct-zol^TM^ RNA MiniPrep kit from QIAGEN. Ficoll-Hypaque (FicoLite H) was obtained from Linarisblue (Wertheim-Bettingen, Germany) and BD FACS^TM^ lysing solution from BD Bioscience. Recombinant human IL-12, IL-4, IL-1β, IL-23, IL-6, TGF-β, neutralizing antibodies against IFNγ and IL-4, True-Nuclear™ transcription factor buffer set, LEGEND MAX™ Human IL-17A, IL-17F and LEGEND MAX™ Free Active TGF-β ELISA kit were obtained from BioLegend (San Diego, USA).

Antibodies used in this study were specific for the following molecules: CD3 (clone OKT3, mouse mAb), CD28 (clone 28.2, mouse mAb) and isotype control antibodies IgG1, κ and IgG2a, κ (mouse mAb, BD Biosciences, Heidelberg, Germany), GAPDH (clone 6C5, mouse mAb, Invitrogen; Eugene, USA), actin (rabbit polyclonal, Sigma-Aldrich, Hamburg, Germany), GSH (clone D8, mouse mAb, Santa Cruz Biotechnology), HIF-1α (rabbit polyclonal, Cayman chemical, USA). For the secondary antibodies, IRDye® 680CW donkey anti-mouse and IRDye® 800CW donkey anti-rabbit were purchased from LICOR Biosciences (Lincoln, USA). Goat anti-mouse IgG+IgM used as coating antibody was from Jackson ImmunoResearch (West Grove, USA). 7-AAD and all fluorescently-labeled antibodies were obtained from BD Biosciences.

### Primary Human T Cell Preparation and Cell Culture

Human peripheral blood mononuclear cells (PBMCs) were obtained from heparinized blood of voluntary healthy donors by density-gradient centrifugation using Ficoll-Hypaque. Pan T cells or naïve CD4^+^ T cells were isolated by negative selection using the pan T cell isolation kit or naïve CD4^+^ T cells isolation kit from Miltenyi Biotec (Bergisch Gladbach, Germany), respectively, according to the manufacturer's instructions. The purity of Pan T cells reached more than 99.5% (Supplementary Figure 1a), of naïve CD4^+^ T cells 99% (Supplementary Figure 1b). The purified T cells were adjusted to 3 x 10^6^ cells/ml in RPMI complete medium (RPMI 1640+10% FBS) and cultured in incubator at 5% CO_2_ and 37°C until use. To costimulate human peripheral blood T cells (PBTs), goat anti-mouse IgG+IgM antibody was used to pre-coat microplates (Nunc, Wiesbaden, Germany), followed by blocking with RPMI complete medium and coating with anti-CD3 (20 ng/mL)/anti-CD28 (5 μg/mL) antibodies or the respective isotype controls. T cells were spun down on the antibodies and incubated at 5% CO_2_ and 37°C for the indicated time points. This study was approved by the Ethics Committee of the Heidelberg University (S-269/2015). The human T cell leukemia cell line Jurkat ACC282 and Raji cells, which served as antigen-presenting cells (APC) were cultured in RPMI complete medium at 5% CO_2_ and 37°C, and cells were split 1:5 every second day by replacing the medium.

### Sampling of Blood From Patients

Peripheral blood from patients with different diseases was collected into sterile heparinized tubes under aseptic conditions. Informed consent for use of the cells was obtained from all patients included in this study. To test the effects of PL on the lymphocytes in whole blood, 100 μl of blood were diluted 1:1 with RPMI medium, blood was treated with DMSO or with 5 μM PL and activated with 1 μg/ml staphylococcal enterotoxin B (SEB) for 20 hours (h). Afterwards, Brefeldin A (10 μg/ml) was used to prevent the secretion of cytokines (29). After 4 h incubation, red blood cells were lysed with BD FACS Lysis Solution for 10 min at room temperature (RT) and simultaneously, white blood cells were fixed. Samples were then washed with FACS wash buffer (FW, PBS containing 0.5% BSA, 0.5% FBS, and 0.07% NaN_3_), permeabilized with FWS (FW containing 0.1% Saponin) and stained with fluorescent-labeled antibodies against CD3, CD69, CD25, IFNγ, and IL-2 in FWS for 20 min at RT. Thereafter, samples were washed and assessed by flow cytometry (LSRII, BD Bioscience, Heidelberg, Germany). Data were analyzed with FlowJo X (FlowJo LLC, Ashland, OR, USA). This study was approved by the Ethics Committee of the Heidelberg University (S-119/2017).

### Cell Viability Assay

Primary human T cells and Jurkat cells were cultured in 200 μL RPMI complete medium with or without 5 μM PL for the indicated time points (5% CO_2_ and 37°C). PL was solved in DMSO, therefore cells were treated with equal concentrations of DMSO as a control. The cells were then washed once with PBS, stained with AnnexinV and 7-AAD in Annexin binding buffer for 20 min at RT. Thereafter, cells were washed once with Annexin binding buffer and analyzed by flow cytometry.

### Measurement of Intracellular ROS and GSH Levels

ROS detection reagent CM-H_2_DCFDA was used for the measurement of intracellular ROS levels and ThiolTracker^TM^ violet dye was used for the measurement of intracellular GSH levels as described before (30). Briefly, for the intracellular ROS measurement, T cells (1 × 10^6^ cells/ml) or Jurkat cells (3 × 10^5^ cells/ml) were washed with PBS and stained with CM-H_2_DCFDA (5 μM) in PBS for 15 min at 37°C. Cells were then washed with PBS, resuspended in RPMI complete medium and treated with DMSO, PL or H_2_O_2_. After the indicated time points, the mean fluorescence intensity (MFI) of the CM-H_2_DCFDA signal was determined by flow cytometry. For the intracellular GSH measurement, T cells (1 × 10^6^ cells/ml) or Jurkat cells (3 × 10^5^ cells/ml) were incubated with DMSO or PL in RPMI complete medium for 1 h or 24 h. Afterwards, cells were rinsed with PBS and stained with 1 μM ThiolTracker^TM^ violet dye (diluted in PBS) for 15 min at 37°C. After washing with PBS, cells were resuspended in PBS and measured immediately by flow cytometry.

### Detection of S-glutathionylation

Western blotting was used for the detection of S-glutathionylated proteins. Control or PL-treated T cells or Jurkat cells were washed with PBS and postnuclear lysates (cytoplasm) were generated. Therefore, T cells were lysed with TKM lysis buffer on ice for 20 min, the nuclei and the cell debris were removed by centrifugation at 14,000 g for 10 min. Proteins in the cytoplasmic fraction were separated by electrophoresis in denaturing SDS polyacrylamide gels (SDS-PAGE), proteins were transferred onto PVDF-membranes and the membranes were blocked in blocking buffer for 1 h. Afterwards, membranes were probed with primary antibodies against GSH (1:200), Actin (1:1,000), GAPDH (1:10,000), and respective IRDye fluorescent-labeled secondary antibodies (1:10,000). The membranes were scanned by a Licor infrared scanner (LI-COR Biosciences, Germany).

The S-glutathionylated protein was further analyzed by mass spectrometry. To this end, postnuclear cell lysates from control or PL-treated T cells (3 × 10^6^ cells/sample), were run on SDS-PAGE and subjected to Coomassie-staining. The bands corresponding to the 42-kDa protein were cut out and analyzed by mass spectrometry. Protein digestion and LC-MS measurement were done as described elsewhere (31). Briefly, gel pieces were washed, dehydrated and incubated with trypsin solution (Thermo-Fisher, Rockford, USA) for 4 h at 37°C. The reaction was quenched by adding of 20 μL of 0.1% trifluoroacetic acid (TFA; Biosolve, Netherlands). The supernatant was dried in a vacuum concentrator before LC-MS analysis. Nanoflow LC-MS analysis was performed with a NanoAcquity UPLC liquid chromatography (Waters, Eschborn, Germany) system coupled to an Orbitrap XL or with an Ultimate 3000 liquid chromatography system coupled to an Orbitrap Q Exactive mass spectrometer (Thermo-Fischer, Bremen, Germany). Detailed information for the LC-MS measurements is described in the Supplementary Material. For the protein identification, raw files were analyzed using Proteome Discoverer with the Sequest version 2.3 (Thermo Fisher Scientific, USA). Sequest was set up to search against Uniprot human databases (retrieved in June, 2017) with trypsin as the digestion enzyme with maximum two missed cleavages. A parent ion mass tolerance was set to 10 ppm and a fragment ion mass tolerance was set to 0.50 Da or 0.02 Da for measurements on the Orbitrap XL or the Q Exactive mass spectrometer, respectively. Proteome Discoverer results were used for a spectral library generation in the Skyline (32) using BiblioSpec algorithm for MS^1^ full-scan quantification of glutathionylated peptides and their unmodified counterparts as previously described (33). After raw file data import into Skyline, chromatographic traces for top three isotopic peaks were manually inspected for proper peak picking of MS^1^ filtered peptides and peak area determination. The summed area of three isotopic peaks was used to calculate peptide intensities. Target peptide areas were normalized to summed area of selected actin peptides (AVFPSIVGRPR; RGILTLK; VAPEEHPVLLTEAPLNPK; SYELPDGQVITIGNER).

### Measurement of Intracellular Calcium

Control or PL-treated T cells (3.5 × 10^6^ cells/sample, 1 × 10^6^ cells/ml) were centrifuged and re-suspended in 250 μL RPMI medium (+2% FBS) with the calcium (Ca^2+^) -binding dye, Indo-1, at a concentration of 2 μg/ml. Cells were then stained at 37°C for 45 min. The dye changes its emission wavelength when binding to Ca^2+^, so a ratio between bound and unbound dye can be calculated. The samples were washed twice with PBS, then resuspended in 500 μL RPMI complete medium. The kinetics of the Ca^2+^ signal was measured by flow cytometry (Fortessa, BD Bioscience, Heidelberg, Germany). To this end, the baseline Ca^2+^ signal was acquired for 1 min, followed by adding anti-CD3 antibody to a final concentration of 20 ng/ml. The Ca^2+^ signal was acquired for another 8 min, until goat-anti-mouse antibody was added (7.2 μg/mL) for crosslinking of the anti-CD3 antibody to induce activation and Ca^2+^ influx to the cytosol. Thereafter, the Ca^2+^ signal was monitored for up to 18 min and then ionomycin was added to induce a maximum Ca^2+^ signal as a positive control and the measurement was stopped after 20 min.

### T Cell/APC Conjugate and Immune Synapse Formation

Conjugates were formed between T cells and Raji cells as described below. T cells (1 × 10^6^ cells/ml) were preincubated with or without 5 μM PL for 1 h or overnight and Raji cells were loaded with 5 μg/ml SEB or kept unloaded. Pretreated T cells and Raji cells were then coincubated for 45 min at 37°C at a ratio of 1:1. Afterwards, 1.5% PFA was added while vortexing the sample for 10 s in order to separate non-specifically bound cells. After 10 min fixation, cells were washed twice with FW buffer followed by staining with anti-CD19 PE-Cy5 and anti-CD3 PE conjugated antibodies. 1 × 10^4^ T cells per sample of three independent experiments were acquired to quantify the proportion of T cell/APC conjugates. CD3 and CD19 double positive events were counted as cell couples.

Immune synapse formation was assessed by multispectral imaging flow cytometry (MIFC, IS100, Amnis Corp., Seattle, WA, USA). To this end, T cell/APC conjugates were formed and fixed as described above. The samples were then stained with anti-CD3 PE-Cy7 and anti-CD18 PE. After permeabilization with FWS buffer, samples were further stained with SiR-actin and DAPI. Thereafter, cells were subjected to MIFC and as many as 15,000 images were acquired per sample. The IDEAS 6.0 software (Amnis, Seattle, WA, USA) was used to analyze the subcellular localization of proteins (34).

### T Cell Activation

T cells (1 × 10^6^ cells/ml) were preincubated with DMSO or different concentrations of PL for 30 min and were seeded in 96-well microplates coated with anti-CD3/CD28 antibodies. In parallel, mouse anti-human IgG1, κ, and IgG2a, κ were coated as isotype control. Then the cells were incubated at 37°C for 24 h and the activation state of the T cells was determined by measuring the expression of CD69 and CD25. Briefly, T cells were collected after plate stimulation and washed twice with FW buffer, fixed with 1.5% PFA for 10 min and stained at RT with anti-CD69 PE-Cy7 and anti-CD25 APC antibodies in FW buffer. After 20 min of staining, cells were washed with FW and measured by flow cytometry. For the detection of the total extra- and intracellular CD69 and CD25, T cells were permeabilized by FWS buffer prior to staining with antibodies in FWS buffer.

### T Cell Proliferation

T cells were washed with PBS, resuspended in PBS containing 1 μM CFSE and incubated at 37°C for 15 min. The unbound CFSE was then removed by washing twice with PBS. T cells were then resuspended in RPMI complete medium and treated with DMSO or PL for 30 min. Afterwards, the cells were seeded on a 96-well microplate coated with anti-CD3/CD28 antibodies and incubated for 72 h. The CFSE signal was determined using flow cytometry and data were analyzed with FlowJo X. The proliferation index was calculated as described previously (30).

### Co-stimulation Induced Cytokine Production

For the assessment of costimulation induced intracellular cytokines, Pan T cells were treated with various concentrations of PL in the presence and absence of exogenously added NAC and then costimulated for 48 h. Afterwards, T cells were restimulated with 10 nM PMA and 1 μg/ml ionomycin for 4 h to enhance the cytokine production. Simultaneously, 10 μg/ml Brefeldin A was used to prevent the secretion of cytokines. Then, T cells were collected and washed twice with FW, fixed in 1.5% PFA for 10 min and permeabilized with FWS for 20 min. Afterwards, T cells were stained with anti-IL-2, anti-IFNγ and anti-TNFα (50 μL/sample) antibodies diluted by FWS for 30 min. Florescence Minus One (FMO) staining was included in control samples to detect unspecific staining. Cells were then washed twice with FWS and measured by flow cytometry. Data were analyzed with FlowJo X.

### Gene Expression Profiling

T cells (1 × 10^6^ cells/sample) were pretreated with DMSO or 5 μM PL for 30 min and then stimulated for 4 h in anti-CD3/CD28 antibodies coated plates. Total RNA was extracted from T cells by trizol. The RNA yield from each sample was detected using a NanoDrop^TM^ 2000 spectrophotometer (Thermo Fisher Scientific, Wilmington, DE), RNA integrity was confirmed using a 2100 Bioanalyzer (Agilent Technologies, Santa Clara, CA). The gene expression profiling was performed with 25 ng total RNA per sample and all RNA samples were analyzed using the Nanostring nCounter GX Human Immunology v2 kit. The gene expression profiling and data analyses were performed as previously described (30). Briefly, the hybridization reaction was carried out at 65°C overnight by mixing RNA samples with nCounter reporter probes and capture probes in hybridization buffer. After hybridization of the probes with the targets of interest, samples were purified and immobilized on a cartridge and data assessed on the nCounter *SPRINT* Profiler. The data were then collected with an automated fluorescence microscope and nCounter digital analyzer, and subsequently assessed with the help of the nSolver Analysis Software Version 4.0 (NanoString Technologies). For the data analysis, sample counts were initially normalized by scaling all the values by the ratios of geometric mean (GM) of sample controls to the overall GM of control gene counts across all samples. This was done for both spike-in positive controls and housekeeping genes. The final counts of the mRNA transcripts were used for the further evaluation.

### Naïve CD4^+^ T Cell Differentiation

Naïve CD4^+^ T cells were isolated as described above. Cells (2 × 10^5^ cells/ml) were treated with DMSO or 1 μM PL and seeded in a 12 well plate coated with anti-CD3/CD28 antibodies. Naïve CD4^+^ T cells differentiation was induced by adding the following cytokines and antibodies. T_H_1: IL-12 (2 ng/ml), anti-IL-4 antibody (2 μg/ml); T_H_2: IL-4 (2.5 ng/ml), anti-IFNγ antibody (2 μg/ml); T_H_17: IL-6 (10 ng/ml), TGF-β (2.5 ng/ml), IL-1β (10 ng/ml), IL-23 (20 ng/ml), anti-IFNγ (2 μg/ml), and anti-IL-4 antibody (2 μg/ml); T_reg_: IL-2 (20 U/ml), TGF-β (5 ng/ml), anti-IFNγ (2 μg/ml) and anti-IL-4 antibody (2 μg/ml). After stimulation and differentiation for 6 days, cells were collected and washed twice with FW buffer, then stained with antibodies specific for the surface molecules CXCR3 (CD183), CCR4 (CD194), and CCR6 (CD196) as mentioned above. The T_H_ subtypes were identified according to the surface staining as T_H_1 cells are CXCR3^+^CCR4^−^CCR6^−^, T_H_2 cells are CXCR3^−^CCR4^+^CCR6^−^ and T_H_17 cells are CXCR3^−^CCR4^+^CCR6^+^ according to standard flow cytometry procedures (35). The T_reg_ cell population was determined by the expression of transcription factor FOXP3. For the FOXP3 staining, cells (1 × 10^6^ cells/sample) were fixed with 100 μL 1 × True-Nuclear™ fixation buffer for 1 h, followed by three times washing with 200 μL 1 × True-Nuclear™ Perm buffer. Afterwards, cells were resuspended in 50 μL 1 × True-Nuclear™ Perm buffer with anti-FOXP3 (1:20) antibody. After incubation for 30 min at RT, cells were washed three times with 200 μL of 1 × True-Nuclear™ Perm buffer, resuspended in 200 μL PBS and measured with flow cytometry.

For the measurement of cytokine production after 6 days of T cell differentiation, cells were centrifuged, cell free supernatants were collected and immediately aliquoted and stored at −80°C. Secreted cytokines in undiluted cell culture supernatants were detected by ELISA kits specific for IL-17A, IL-17F, and TGF-β as per the manufacturer's instructions.

### HIF-1α Detection

Western blot was used to detect HIF-1α. Briefly, T cells (1 × 10^6^ cells/ml) were treated with DMSO or PL as indicated, and activated in anti-CD3/CD28 antibodies coated plates for 24 h. Then, the cells were collected, washed with PBS and total lysate was prepared using PBS with 1 × reducing sample buffer. Proteins were detected by western blotting as described above with the following antibodies: HIF-1α (1:200), Actin (1:1,000) and respective IRDye® conjugated secondary antibodies (1:10,000).

### Statistics

Statistical tests were performed with GraphPad Prism 6.0 (GraphPad; San Diego, USA). Two data sets were analyzed using *t*-test or paired *t*-test for matched observations. Multiple groups were compared using ANOVA. All data are presented as mean ± standard error of the mean (SEM). *P* < 0.05 were considered statistically significant.

## Results

### PL Induces a Prooxidative Milieu in Primary Human T Cells

First, we have determined whether PL is toxic to human T cells. To this end, we prepared freshly isolated human PBTs and assessed T cell viability after treatment with 10 μM PL up to 24 h. Since, it was reported that 1511.9 ng/ml (4.8 μM) of PL could be found in rat plasma after 1 dose of 50 mg/kg PL intraperitoneal injection (36), in our study we considered concentrations of PL up to 10 μM as physiologically relevant. Even the highest PL concentration used in our experiments (10 μM) showed no significant cytotoxicity on primary human T cells (Supplementary Figure 2a). Interestingly, this concentration of PL induced cell death in malignant Jurkat T leukemia cells over the same time period (Supplementary Figure 2b) which is in line with previous studies showing cytotoxic effects of 10 μM PL on myeloid and B cell leukemia cells (37, 38).

**Figure 1:**
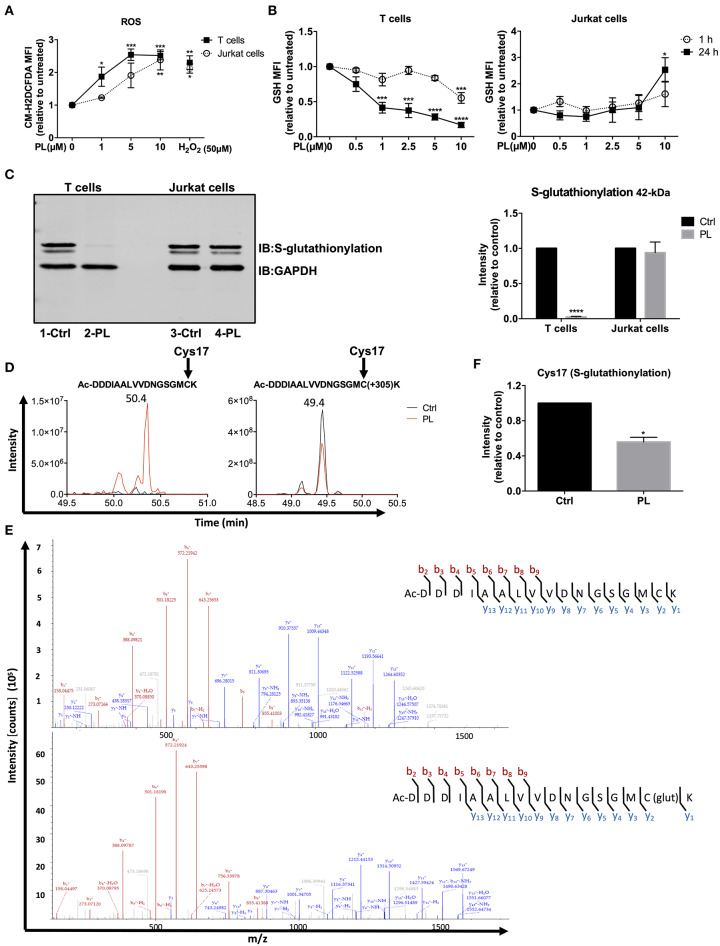
PL induces a prooxidative state in primary human T cells. **(A)** The intracellular ROS levels were assessed by flow cytometry. Primary human T cells (black squares) and Jurkat cells (open circles) were preloaded with CM-H_2_DCFDA and, thereafter, treated for 1 h with or without PL as indicated (*n* = 3; mean; SEM; *p < 0.05, **p < 0.01, ***p < 0.001). **(B)** Intracellular GSH levels of T cells (left panel) and Jurkat cells (right panel) were assessed by flow cytometry. Cells were treated with or without PL as indicated for 1 h (open circle) or 24 h (black square) and stained with ThiolTracker^TM^ violet dye for 15 min (*n* = 3; mean; SEM; *p < 0.05, ***p < 0.001, ****p < 0.0001). **(C)** Protein S-glutathionylation was detected by western blotting. Representative immunoblot (left) shows the S-glutathionylated 42-kDa protein and GAPDH in control or 5 μM PL-treated T cells (lane 1, 2) and Jurkat cells (lane 3, 4). Bar graph (right) shows the quantitative analysis of three different experiments. S-glutathionylated 42-kDa protein levels were related to the expression levels of GAPDH. Values were normalized to the control, which was set as 1 (*n* = 3; mean; SEM; ****p < 0.0001). **(D)** Elution profile of acetylated N-terminal peptide Ac-DDDIAALVVDNGSGMCK of actin in control (black) or PL-treated (red) T cells in its unmodified (left) and glutathionylated (+305) form (right). Shown are the representative extracted ion chromatograms from three biological replicates. **(E)** MS^2^ spectrum of unmodified (upper graph) and glutathionylated (lower graph) showing N-terminal acetylated Cys17 identified at 50.4 min and 49.4 min, respectively. **(F)** Quantification of S-glutathionylated Cys17 under control and PL treatment conditions. Values were normalized to the control, which was set as 1 (*n* = 3; mean; SEM; *p < 0.05).

PL exhibits potent anticancer activity in multiple cancer types by increasing the cellular ROS levels (39). ROS are also important regulators of T cell activation and function (24, 40, 41). The impact of PL on the redox system of primary human T cells, however, is unknown. We therefore evaluated the intracellular ROS levels in primary human T cells after PL treatment. To this end, the ROS-sensitive probe, CM-H_2_DCFDA, was used to stain T cells, followed by T cell treatment with 1–10 μM PL for 1 h prior to flow cytometric analysis. Supplementary Figure 3a shows the gating of lymphocytes and the staining with CM-H_2_DCFDA. PL treatment induced a significant increase of ROS in a concentration-dependent manner (Figure 1A). The intracellular ROS levels in primary human T cells after treatment with 5–10 μM PL were comparable to those observed after treatment with 50 μM H_2_O_2_. Similar to primary human T cells, the ROS levels in Jurkat leukemia cells also increased significantly after PL treatment (Figure 1A).

The response of cells to prooxidative substances is antagonized by antioxidant systems, primarily by GSH. Therefore, we next determined the intracellular levels of GSH, an important ROS-scavenger, using ThiolTracker^TM^ violet dye in both primary human T cells and Jurkat cells. Supplementary Figure 3b shows the gating of lymphocytes and the staining with ThiolTracker^TM^. After 1 h treatment, 10 μM PL was able to significantly diminish GSH levels, whereas already 1 μM PL was sufficient to significantly decrease the amount of GSH in T cells after 24 h treatment (Figure 1B, left). In contrast, the GSH levels in Jurkat cells were even increased after 24 h treatment with 10 μM PL (Figure 1B, right).

GSH can form mixed disulfides with oxidized cysteine residues in proteins in response to mild oxidative stress, which is named S-glutathionylation. This process is considered as a defense mechanism to protect proteins from irreversible oxidative states (42). Since PL shifted the cellular redox equilibrium toward prooxidative conditions, we next investigated whether PL interfered with the S-glutathionylation of cellular proteins. For this purpose, we treated the cells with 5 μM PL for up to 4 h. Subsequently, cell lysates were loaded on SDS-PAGE and immunostained for S-glutathionylation using a monoclonal anti-GSH antibody. We observed a strong glutathionylation signal of a 42-kDa protein which decreased strongly (deglutathionylation) in T cells treated with PL (Figure 1C, lane 1-Ctrl and 2-PL). Notably, treatment of Jurkat cells with PL did not change the S-glutathionylation of the same protein band (Figure 1C, lane 3-Ctrl and 4-PL). This finding is consistent with the strongly diminished intracellular levels of GSH, the essential material for S-glutathionylation, only in PL-treated T cells but not in Jurkat cells.

The 42-kDa protein was further analyzed by liquid chromatography-tandem mass spectrometry (LC-MS^2^). LC-MS^2^ analysis revealed that the highly S-glutathionylated 42-kDa band was beta-actin. To further elaborate this finding, we inspected which cysteines in actin were S-glutathionylated and whether PL treatment alters the relative levels of this modification. To this end, we searched for unmodified, S-glutathionylated (+305), sulfenylated (SOH), sulfinylated (SO_2_H), and sulfonylated (SO_3_H) forms of the cysteine-containing peptides. The peptide Ac-DDDIAALVVDNGSGMCK was detected as unmodified, S-glutathionylated (+305), sulfinylated (SO_2_H, +32) and sulfonylated (SO_3_H, +48) on Cys17 with MS^2^ spectra at 50.4, 49.4, 50, and 50 min, respectively (Figure 1D and Supplementary Figures 4a,c). The MS^2^ spectrum fingerprint for all peptides is comparable. *b* ions have the same masses and relative intensities in all spectra. Relative intensities of *y* ions are comparable in all spectra while their masses are (except for *y*_1_) 305.0681, 31.990 or 47.986 m/z higher in the spectrum of the S-glutathionylated, sulfinylated or sulfonylated peptide, respectively (Figure 1E and Supplementary Figures 4b,d). These MS^2^ spectra show that Cys17 is present as unmodified, S-glutathionylated (+305), sulfinylated (SO_2_H) and sulfonylated (SO_3_H) residue in the peptide Ac-DDDIAALVVDNGSGMCK.

For relative quantification of the peptides, peak areas were calculated with the Skyline software (32) and non-cysteine-containing peptides were used for normalization in each sample. The unmodified peptide Ac-DDDIAALVVDNGSGMCK was detected in the PL-treated sample, whereas it was absent in the control (Figure 1D, left). Cys17 glutathionylated peptide Ac-DDDIAALVVDNGSGMC(+305)K was detected with intense signals in both control and PL-treated samples (Figure 1D, right). Figure 1F shows that the intensity of the S-glutathionylated peptide (Cys17) decreased significantly upon PL treatment compared to the control sample. Moreover, lower levels of S-glutathionylation on Cys217 upon PL treatment were also found in two experiments, while it was not detected in the third experiment in both control and PL-treated samples (data not shown). Reasonably, sulfinylated (SO_2_H) and sulfonylated (SO_3_H) forms of the Cys17 in peptide Ac-DDDIAALVVDNGSGMCK in control and PL-treated sample were detected, and the signal intensities increased for both peptides with PL treatment (Supplementary Figures 4a,c,e). This indicates oxidation of the cysteine to sulfinic or sulfonic acid.

Taken together, PL exerts prooxidative activity on primary human T cells, based on an increase in ROS levels and a decrease in GSH levels. In Jurkat cells, PL also enhanced the intracellular ROS levels, but the levels of GSH remained stable or rather increased. In line with these observations, PL diminished the S-glutathionylation of actin in T cells but not in Jurkat cells.

### PL Inhibits Immune Synapse Formation Between APC and T Cells

Since excessive ROS production or prolonged exposure to high ROS levels impair T cell functions (24), we investigated whether PL influences T cell activation and T cell-dependent immune responses.

**Figure 2:**
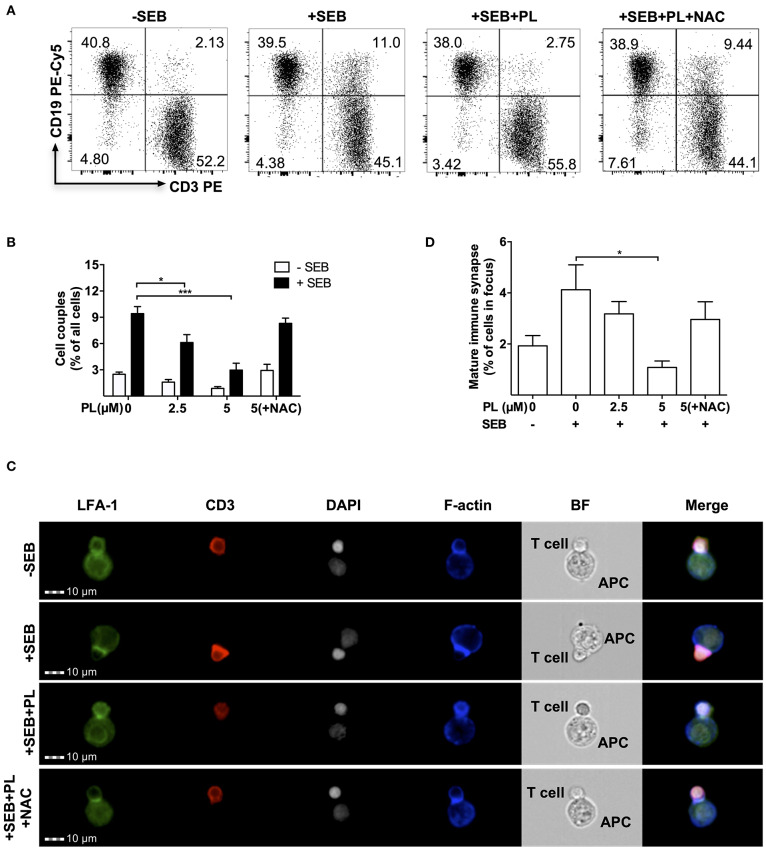
PL interferes with T cell/APC couple formation and immune synapse maturation. **(A)** Dot plots of T cell/APC couples. Formation of couples was assessed by flow cytometry via CD3 and CD19 staining. T cells were treated overnight without or with 5 μM PL or 5 μM PL +NAC (3 mM) prior to incubation with APCs in the absence or presence of SEB. The dot plots are representative for three experiments. **(B)** Quantification of the couple formation between T cells and APCs (*n* = 3; mean; SEM; *p < 0.05, ***p < 0.001). **(C)** Formation of mature immune synapses was analyzed by multispectral imaging flow cytometry (MIFC). Cells were stained for LFA-1 (green), CD3 (red), nuclei (DAPI, white), and F-actin (blue). BF represents the Bright Field image. The merged images show the digital overlay of all four colors. Images are representative for three experiments. **(D)** Quantification of mature immune synapses between T cells and APCs (*n* = 3; mean; SEM; *p < 0.05).

In a physiological setting, one of the first steps of T cell activation is the formation of a contact zone with an APC and the maturation of the immunological synapse (IS). To investigate this, control or PL-pretreated T cells (1 h or overnight pretreatment with 5 μM PL) were cultured with SEB-loaded Raji cells which served as APCs. First, T cell/APC conjugates were identified by flow cytometry (Figures 2A,B). As expected, in the absence of SEB, only 2.5 ± 0.25% T cell/APC conjugates were found, whereas this number of conjugates increased to 9.4 ± 0.8% in the presence of SEB. No effect on cell couple formation was observed after 1 h preincubation with 5 μM PL (data not shown), but overnight pretreatment with PL significantly decreased the formation of T cell/APC conjugates in the presence of SEB to 3.0 ± 0.79% (Figure 2B). In order to clarify whether this result is due to the prooxidative capacity of PL described above (compare Figure 1), we made use of the antioxidant NAC. As a synthetic precursor of intracellular cysteine and GSH, NAC can lead to replenishment of GSH stores (43). Intriguingly, we observed that NAC (3 mM) could prevent the PL-induced downregulation of T cell/APC conjugates formation (Figure 2B).

The impact of PL on formation of a mature immune synapse was investigated by multi-spectral imaging flow cytometry (MIFC). By defining regions of interest, MIFC allows the spatial quantification of the accumulation of receptors at the IS. T cell/APC couples were identified according to staining of nuclei with DAPI and detection of CD3 expression in T cells. The mature IS was then defined by accumulation of LFA-1 (green) and CD3 (red) in the T cell/APC contact area. As expected, while most T cells did not show LFA-1 and CD3 enrichment in the contact area without SEB treatment, a clear receptor enrichment -hence IS maturation- was observed in the presence of SEB. In samples pretreated overnight with PL, the enrichment of LFA-1 and CD3 in the contact zone was very low but it occurred normally when T cells were preincubated with PL and NAC (Figures 2C,D). Together, overnight pretreatment of T cells with PL impairs the formation of a mature IS between T cells and APCs, most likely due to depletion of GSH.

### PL Inhibits T Cell Calcium Signaling

**Figure 3:**
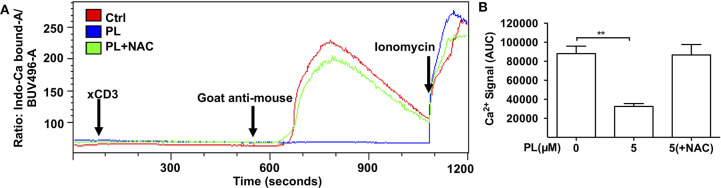
PL inhibits TCR-mediated Ca^2+^ flux. **(A)** Ca^2+^ flux in control (red), 5 μM PL (blue) or 5 μM PL+NAC (green) pretreated (overnight) T cells was measured using flow cytometry. Cells were stimulated by adding CD3 antibodies (xCD3) followed by crosslinking with goat anti-mouse antibodies as indicated. Ionomycin was added as a positive control at the end of each measurement. The depicted graphs are representative for three independent experiments. **(B)** Quantification of Ca^2+^ signal as the area under the curve (AUC) (*n* = 3; mean; SEM; **p < 0.01).

IS formation initiates and controls Ca^2+^ signals in T cells (44). We asked whether PL also influences the anti-TCR/CD3-induced Ca^2+^ signaling which occurs independently of T cell/APC conjugate formation. Figure 3A shows the Ca^2+^ flux signal monitored by flow cytometry. The parameter AUC (area under curve) was used to evaluate the Ca^2+^ signal in our experiments, which refers to the total amount of cytosolic Ca^2+^ during the whole recorded period of Ca^2+^ flux. These data revealed that the Ca^2+^ signal in control T cells increased steeply after CD3 cross-linking while overnight pretreatment of T cells with 5 μM PL inhibited the TCR/CD3-mediated Ca^2+^ flux. This inhibition was completely prevented by NAC (Figures 3A,B). Together, these results imply that PL also interferes with Ca^2+^ signaling in T cells by depleting GSH.

### PL Inhibits Costimulation-Induced CD69 and CD25 Expression in Primary Human T Cells

**Figure 4:**
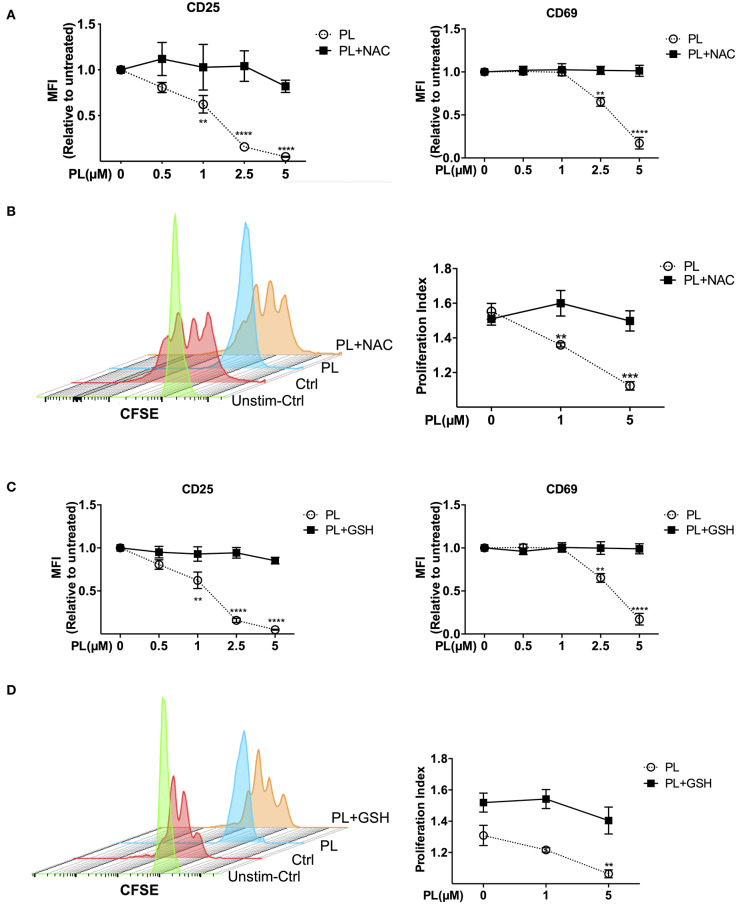
Immunosuppressive effects of PL were abrogated by thiol-containing antioxidants. **(A,C)** Expression of CD25 (left) and CD69 (right) in T cells was analyzed by flow cytometry. T cells in the presence/absence of PL and **(A)** NAC or **(C)** GSH were costimulated with anti-CD3/CD28 antibodies for 24 h. Shown are the MFI ratios of PL-treated to untreated samples. **(B,D)** T cell proliferation was detected via staining of CFSE. T cells were loaded with CFSE, thereafter costimulated with anti-CD3/CD28 antibodies in the absence/presence of PL without or with addition of exogenous **(B)** NAC or **(D)** GSH. CFSE signal was measured after 72 h of costimulation by flow cytometry. Shown are representative histograms (left) and the proliferation index (right) (*n* = 3; mean; SEM; **p < 0.01, ***p < 0.001, ****p < 0.0001).

To investigate whether pretreatment with PL for 30 mins interferes with later T cell activation events, expression of the T cell activation markers CD69 and CD25 in primary human T cells was assessed after costimulation with anti-CD3/CD28 antibodies or isotype control antibodies. The flow cytometry analysis procedure is shown in Supplementary Figure 5a. T cells seeded in isotype control antibody-coated plates are similar to resting T cells according to the CD69 and CD25 surface staining (Supplementary Figure 5b). After costimulation with anti-CD3/CD28 antibodies for 24 h, CD69 and CD25 were highly expressed on the T cell surface in the absence of PL, while in the presence of PL, their expression was suppressed in a dose-dependent manner (Figure 4A and Supplementary Figure 5c). Intriguingly, while the MFI value of CD69 was decreased after PL treatment, the percentage of CD69-positive cells was not altered under the same conditions. Moreover, not only the surface expression, but also the total amount of CD69 and CD25 within the cells was decreased upon PL treatment (Supplementary Figure 5d). This indicates that PL inhibited the expression of both proteins rather than impairing the receptor transport to the surface. Again, treatment with NAC prevented the PL-induced downregulation of CD69 and CD25 (Figure 4A).

### PL Inhibits Costimulation-Induced Proliferation of Primary Human T Cells

T cell proliferation over time can be monitored via labeling T cells with CFSE. To this end, we pretreated CFSE-labeled T cells with or without PL for 30 mins, and determined T cell proliferation by flow cytometry after 72 h of costimulation. As shown in Figure 4B, compared to untreated control cells, PL treatment significantly inhibited T cell proliferation. Again, NAC abolished the suppressive effect of PL on T cell proliferation. In order to determine whether these immunosuppressive effects are due to the depleted GSH stores, we repeated the experiments with exogenously added GSH. Indeed, inhibition of the expression of CD69 and CD25 (Figure 4C), and T cell proliferation (Figure 4D) were completely prevented in the presence of GSH. All together, these findings demonstrate that PL acts as an immunosuppressive agent on primary human T cells through depletion of intracellular GSH. PL induced immunosuppression can be prevented by the antioxidants GSH or NAC.

### PL Suppresses Expression of Genes Important for T Cell Activation and Proliferation

**Figure 5:**
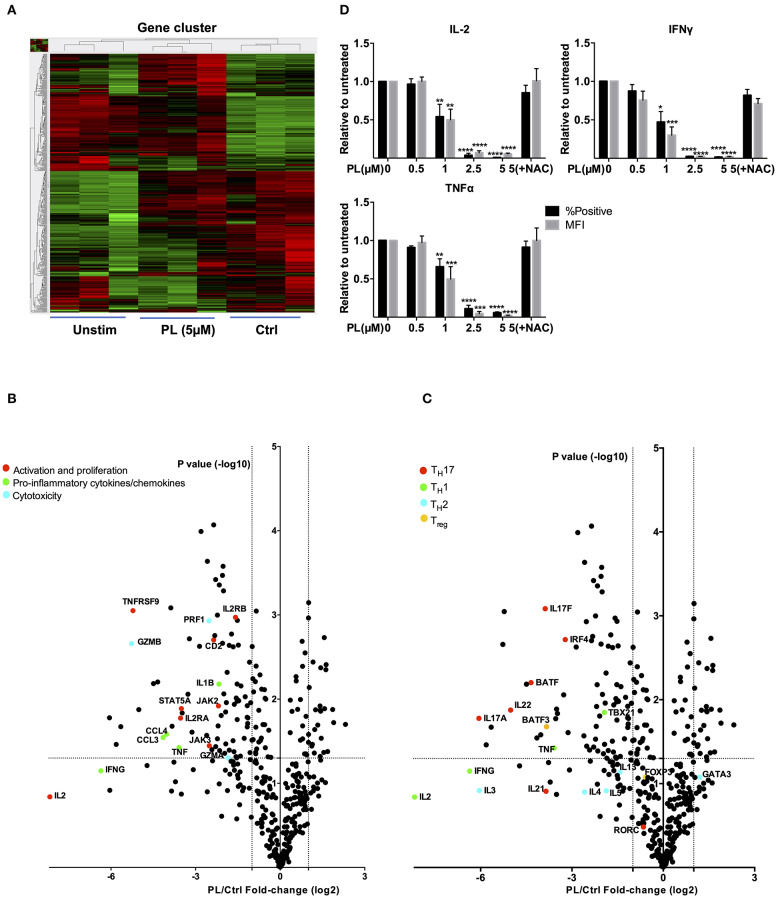
Gene expression analysis (nCounter®) and intracellular cytokine staining. **(A)** Hierarchical clustering of differentially expressed mRNAs in T cells which were unstimulated (Unstim), anti-CD3/CD28 antibodies stimulated with PL (PL 5 μM) or without PL (Ctrl) treatment. Each column represents an individual sample (*n* = 3). Relative change is indicated by the color scale (red: high; green: low). **(B,C)** Volcano plots show differentially expressed genes **(B)** important for T cell activation and cytokines and **(C)** related to T_H_1, T_H_2, T_reg_, and T_H_17 cells. Log_2_ transformed fold-change in expression (PL vs. Control) on the x-axis is plotted against significance [–log10 (*p*-value)] on the y-axis. The horizontal and vertical dashed lines indicate cutoff for significance *p* < 0.05 (−log10 p-value >1.3) and for fold-change ≥2/ ≤ −2, respectively. **(D)** Protein levels of IFNγ, TNFα and IL-2 were detected by intracellular cytokine staining. Shown are ratios of PL-treated cells to the control with regard to the % positive cells (black bars) and MFI (gray bars) (*n* = 3; mean; SEM; *p < 0.05, **p < 0.01, ***p < 0.001, ****p < 0.0001).

Next, we took advantage of the NanoString nCounter® Technology and the GX Human Immunology v2 panel to gain an unbiased view on the expression of differential mRNA profiles. The genes evaluated were related to leukocyte functions including major classes of cytokines and their receptors. The final counts of the mRNA transcripts were visualized by hierarchical clustering (Figure 5A). For these experiments, primary human T cells were again pretreated for 30 min with PL or solvent control and then costimulated with anti-CD3/CD28 antibodies for 4 h. Upon PL treatment, 81 genes were significantly downregulated and 27 genes were significantly upregulated (fold change >2, *p* < 0.05) compared to the control. Notably, consistent with our finding of diminished CD25 (*IL-2RA*) at the protein level (compare Figure 4A), the mRNA level of *IL-2RA* was also substantially decreased by PL treatment (Figure 5B, red dot). Intriguingly, other genes that are important for T cell activation and proliferation were also inhibited upon PL treatment, e.g., *CD2* and the TNF receptor superfamily member 9 [*TNFRSF9, a TNF* receptor that possesses costimulatory activity for activated T cells (45)] and *JAK2, JAK3*, and *STAT5A* [an essential mediator of IL-2 signaling in T cells (46)] (Figure 5B, red dots), as well as the proinflammatory cytokines *IFN*γ and *TNF*α (Figure 5B, green dots). Moreover, the expression of the key proinflammatory cytokine *IL-1B* (47), the chemokine (C-C motif) ligands *(CCL) 3* and *CCL4*, which can be produced by T cells following nonspecific activation or T cell receptor engagement (48) were inhibited by PL treatment (Figure 5B, green dots). In addition, PL was able to suppress the expression of cytolytic enzymes like *Granzyme* (*Grz*) *A, GrzB* and *Perforin*, which are critical mediators of anti-viral and anti-tumor immunity (Figure 5B, blue dots). These results, together with the aforementioned findings, demonstrate that PL is an inhibitor for T cell activation and possesses anti-inflammatory properties. Furthermore, our data imply that PL is a potential suppressor of T cell-mediated cytotoxicity.

Intriguingly, the expression of T_H_17-specific cytokines, e.g., *IL-17A, IL-17F*, and *IL-22* as well as expression of B cell activating transcription factor (*BATF*) (49) and interferon regulatory factor 4 (*IRF4*) (50), which together initiate a transcriptional program for T_H_17 cell development (51), were inhibited by PL treatment (Figure 5C, red dots). Expression of *RORC* gene was, however, unaffected. We have then further checked genes associated with other T cell subpopulations, i.e., T_H_1, T_H_2, and T_reg_ cells. In addition to *TNF*α, the expression of *IL-2* and the T_H_1 cytokine *IFN*γ were inhibited by PL although the differences to untreated control cells were not statistically significant. PL significantly inhibited the T_H_1-specific transcription factor *TBX21* (Figure 5C, green dots). The expression of T_H_2-associated cytokines, e.g., *IL-3, IL-4, IL-5*, and *IL-13* was in part suppressed by PL, but not significantly and to a lower extent compared to T_H_1 cytokines (Figure 5C, sky blue dots). The T_H_2-specific transcription factor *GATA3* was even increased by tendency upon PL treatment (Figure 5C, sky blue dot). Following costimulation for 4 h, the T_reg_-related transcription factor *FOXP3* remained unaffected by PL, but *BATF3*, a transcription factor that prevents the differentiation of T_reg_ cells (52), was inhibited significantly by PL (Figure 5C, yellow dots). These results together provided first hints that PL acts differently on various T cell populations: At the gene expression level, T_H_17-associated cytokines were more sensitive to PL treatment compared to T_H_1 and T_H_2 cytokines (Figure 5C).

To test these findings at the protein level, the cellular content of IL-2, IFNγ, and TNFα was determined by intracellular staining. Consistent with our findings of diminished mRNA levels of *IL-2, IFN*γ, and *TNF*α (Figures 5B,C), also the intracellular protein levels of these cytokines were substantially decreased by PL treatment (Figure 5D). Note that FMO samples showed that there is no nonspecific staining for these cytokines (Supplementary Figure 6a). Also, at the protein level, the PL-mediated decrease of IL-2, IFNγ, and TNFα was prevented when the cells were coincubated with NAC, which is consistent with the hypothesis that depletion of GSH plays a crucial role in PL-mediated T cell immunomodulation. Of note, NAC alone did not upregulate expression of IL-2, IFNγ, and TNFα (Supplementary Figure 6b).

### PL Inhibits the Differentiation of Naïve CD4^+^ T Cells Into T_H_17 Cells While Promoting the Development of T_reg_ Cells

**Figure 6:**
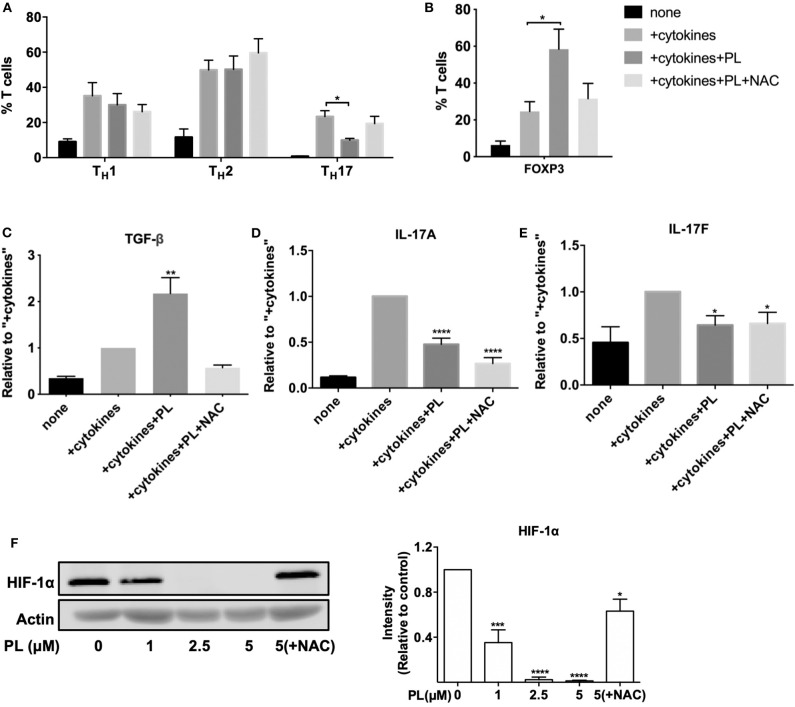
PL inhibits the differentiation of naïve CD4^+^ T cells into T_H_17 cells while promoting the development of T_reg_ cells. **(A–C)** Naïve CD4^+^ T cells were costimulated by anti-CD3/CD28 antibodies either alone (none) or in the presence of T_H_1, T_H_17, or T_H_2 polarizing cytokines without (+cytokines) or with 1 μM PL treatment (+cytokines +PL), or with both PL and NAC treatment (+cytokines +PL +NAC). **(A)** The differentiation of T_H_1, T_H_2, and T_H_17 cells was assessed by surface staining of CD183, CD194, and CD196 and was analyzed by flow cytometry. Shown are the percentage of the respective T_H_ subset in the total T cell population (*n* = 5; mean; SEM; *p < 0.05). **(B)** T_reg_ cells were identified by nuclear staining of FOXP3 (*n* = 3; mean; SEM; *p < 0.05). **(C)** TGF-β levels after T_reg_ polarizing differentiation conditions, **(D)** IL-17A and **(E)** IL-17F levels after T_H_17 polarizing differentiation conditions were detected by ELISA (*n* = 3; mean; SEM; *p < 0.05, **p < 0.01, ****p < 0.0001). **(F)** HIF-1α was detected by western blot (left). T cells were pretreated with or without PL and NAC as indicated for 30 min and seeded into anti-CD3/CD28-coated plates for 24 h. Cell lysates were loaded on SDS-PAGE gel and immunoblotted for HIF-1α and actin. The expression levels of HIF-1α were normalized to the expression levels of actin. The bar graph (right) shows the quantitative analysis of three different experiments (*n* = 3; mean; SEM; *p < 0.05, ***p < 0.001, ****p < 0.0001).

The results from the nCounter gene analysis suggested that PL differentially affects T cell subpopulations. To further investigate this finding, we have next measured the effect of PL on the differentiation of naïve CD4^+^ T cells into T_H_1, T_H_2, T_H_17, and T_reg_ cells. Naïve CD4^+^ T cells were cultured under appropriate polarizing differentiation conditions (+cytokines) in the absence or presence of 1 μM PL (+cytokines+PL) as described in Materials and Methods. After 6 days, the differentiation into T_H_1, T_H_2, and T_H_17 cells was assessed by surface marker staining (Figure 6A and supplementary Figure 7a) and cytokine detection (Figures 6C–E). Nuclear FOXP3 staining was used for identifying T_reg_ cells (Figure 6B and Supplementary Figure 7b). 7-AAD staining confirmed that cells did not die after 6 days of 1 μM PL treatment (data not shown). Quantification of the T cell subpopulations (Figures 6A,B) confirmed that the percentage of T_H_1, T_H_2, T_H_17, and T_reg_ cells in the respective polarizing conditions (+cytokines) was much higher than with anti-CD3/28 costimulation alone (none), which proved that the polarizing conditions were optimal. PL inhibited the differentiation of naïve CD4^+^ T cells into T_H_17 cells while it had no clear effect on the differentiation into T_H_1 and T_H_2 cells, as assessed by staining of the respective T cell surface markers (Figure 6A). Interestingly, FOXP3 staining showed that PL significantly promoted the differentiation of naïve CD4^+^ T cells into T_reg_ cells (Figure 6B). This notion holds true not only for the proportion of FOXP3^+^ T_reg_ cells, but also for the absolute number of FOXP3^+^ T_reg_ cells. Importantly, NAC treatment prevented the PL mediated decrease in the T_H_17 population and the PL mediated increase in the T_reg_ population (+cytokines+PL+NAC) (Figures 6A,B).

To get a more detailed picture about the consequences of PL for the differentiation of naïve CD4^+^ T cells into T_H_17 and T_reg_ cells, we analyzed the levels of the anti-inflammatory T_reg_ cell cytokine TGF-β, and the proinflammatory T_H_17 cell cytokines IL-17A and IL-17F. Indeed, in line with the increased proportion of FOXP3^+^ cells (Figure 6B), under T_reg_ polarizing differentiation conditions the concentration of TGF-β also increased significantly upon treatment with PL (+cytokines+PL) compared to the control without PL (+cytokines). And again, NAC prevented this effect (Figure 6C). As expected, PL treatment (+cytokines+PL) significantly diminished the levels of the proinflammatory T_H_17 cell cytokines IL-17A and IL-17F (Figures 6D,E). Surprisingly, these cytokine levels remained unaffected by NAC.

It was reported that HIF-1α, a subunit of the heterodimeric transcription factor HIF-1, enhances T_H_17 development and attenuates T_reg_ development (53). Intriguingly, we could show by western blot analysis that PL strongly inhibits the expression of HIF-1α in a dose-dependent manner starting already at a concentration of 1 μM PL. This inhibition was prevented by NAC treatment (Figure 6F). Thus, the diminished expression of HIF-1α may provide one molecular explanation for the PL-induced downregulation of T_H_17-related genes. Taken together, PL affects T cell subpopulations differently. It inhibits the differentiation of naïve CD4^+^ T cells into proinflammatory T_H_17 cells, while promoting the differentiation into T_reg_ cells.

### PL Shows Immunosuppressive Effects on Whole Blood Lymphocytes Derived From Patients With Autoimmune Diseases

**Figure 7:**
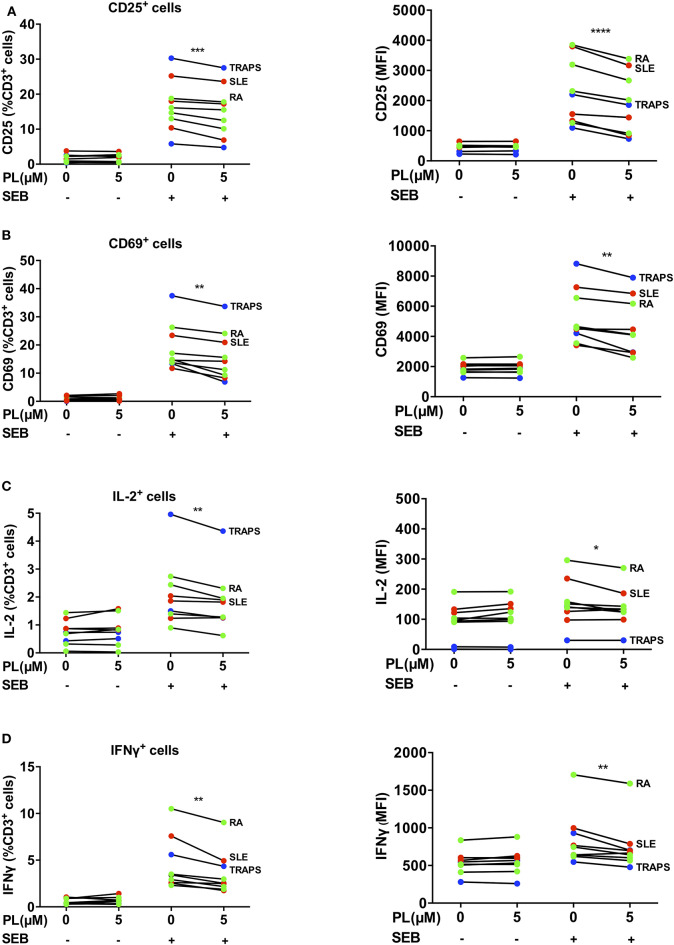
PL downregulates CD69, CD25, IL-2 and IFNγ in lymphocytes of patient-derived whole blood samples**. (A–D)** Whole blood samples from patients with RA (green), SLE (red) and TRAPS (blue) were treated with or without PL and activated with SEB overnight followed by erythrocytes lysis and subsequent immunofluorescence staining and flow cytometry. The CD3 positive cells within the white blood cells were analyzed for expression of **(A)** CD25, **(B)** CD69, **(C)** IL-2, and **(D)** IFNγ. Shown are percent positive T cells (left) and MFI (right). Statistical analysis was conducted using paired *t*-test (*n* = 8; mean; SEM; *p < 0.05, **p < 0.01, ***p < 0.001, ****p < 0.0001).

Given the newly discovered immunomodulatory role of PL on primary human T cells, it was tempting to speculate that PL could be beneficial for patients with autoimmune diseases. Therefore, we analyzed the impact of PL on whole blood samples from patients with RA and systemic lupus erythematosus (SLE), which are prototypical autoimmune rheumatic diseases (54), as well as from patients with TNF receptor-associated periodic syndrome (TRAPS), a very rare autoinflammatory disease. For these analyses, 100 μl of heparin blood were pretreated with 5 μM PL and activated with SEB overnight. Erythrocytes were then lysed and leukocytes were permeabilized, stained with fluorescently-labeled antibodies specific for CD3, CD69, CD25, IFNγ, and IL-2 and assessed by flow cytometry. In the presence of PL, the expression of the T cell activation markers CD25 (Figure 7A, and Supplementary Figure 8) and CD69 (Figure 7B, and Supplementary Figure 8) after SEB stimulation was significantly downregulated in the lymphocyte population (CD3^+^). Consistently, a PL-mediated inhibition was also observed on the production of IL-2 (Figure 7C) and the proinflammatory cytokine IFNγ (Figure 7D). Taken together, this experiment confirmed the immunosuppressive activity of PL on human T cells in clinically relevant samples. This implies that PL might be a promising substance for the treatment of autoimmune diseases.

## Discussion

PL has been of interest as a potential anticancer drug, while its impact on primary human T cells, which are responsible for anti-tumor immune responses, remained unknown. We therefore examined in our study whether PL interferes with the function and differentiation of primary human T cells. While PL decreased ROS in mouse DCs (19) and in human TNFα stimulated FLS of RA patients (23), we have found that PL induces a prooxidative state in primary human PBTs. This manifested as an increased amount of ROS and largely depleted intracellular GSH pools. Moreover, we have revealed here for the first time that PL inhibits the activation and the proliferation of primary human PBTs. Even more important, PL is able to attenuate the development of human proinflammatory T_H_17 cells and to enhance the development of T_reg_. These data uncover a potential of PL to act as a cell type-specific immunomodulator in human and suggest that PL might be a promising substance for treating autoimmune diseases.

The effects of PL on T cell functions can at least partially be explained by the depletion of GSH, since a precursor for GSH biosynthesis, namely NAC, or exogenously added GSH were able to restore T cell functions in the presence of PL. Maintaining optimal GSH levels in T cells is critical for regulating the redox state of proteins, thus keeping the cells in a normal functional state. Depletion of GSH could potentially interfere with the S-glutathionylation, thereby the oxidation state and function of various proteins. Indeed, analysis of protein S-glutathionylation by western blotting and mass spectrometry demonstrated that PL decreased S-glutathionylation of beta-actin. In this context, we have shown that in human T cells Cys17 and Cys217 were highly glutathionylated in the absence of PL. So far, only S-glutathionylation on Cys217 of actin had been described (55). S-glutathionylation on Cys17 of actin is shown for the first time in this study. S-glutathionylation of proteins can change their function in several ways. Intriguingly, we found that when Cys17 was not glutathionylated in PL treated T cells, it existed in higher oxidation states, i.e., sulfinylated or sulfonylated states. This is in line with the notion that GSH protects cysteines from irreversible oxidation (42). Since diminished GSH and diminished protein S-glutathionylation resulting in hyperoxidation of proteins can alter the function of many proteins, these findings provide a potential general mechanistical explanation for the loss of cellular functions upon PL treatment.

While high levels of ROS lead to irreversible protein oxidation, thus loss of cellular function (24), low levels of ROS with readily reversible protein oxidation even promote T cell functions (40). The sensitivity of different proteins and T cell subsets to oxidative stress varies (56). In this context, different T cell subsets may react differentially to the same level of oxidative stress. Immune cell-specific gene expression analysis revealed that the most dampened genes upon PL treatment were T_H_17 cell-related, followed by T_H_1-related genes. Importantly, while PL impaired the development of T_H_17 cells, at the same time it increased the development of T_reg_ cells. This is consistent with the finding that ROS limit T_H_17 cell differentiation (27) but increase T_reg_ cell differentiation (57). HIF-1α is another key factor that controls the T_H_17/T_reg_ balance. Lack of HIF-1α in mice resulted in enhanced T_reg_ development but diminished T_H_17 development and protected the mice from autoimmune neuroinflammation (53, 58). In this context, we found that in human T cells PL inhibited the expression of HIF-1α in a dose-dependent way. This effect was in turn prevented by NAC treatment. Thus, inhibition of HIF-1α may offer an additional explanation for the altered T_H_17/T_reg_ balance under PL treatment.

T_H_1 and T_H_17 cells are involved in the progression of many autoimmune diseases (59, 60). Due to the inhibitory effect on T_H_1 and T_H_17 cells, PL could be helpful for the treatment of these diseases. In line with this assumption, we showed that also in T cells from patients with autoimmune diseases, PL led to reduced expression of the T cell activation markers CD69 and CD25 and diminished production of IFNγ and IL-2. These results are in line with previous findings, which showed that PL inhibits T_H_17 cells in mouse CIA models and reduces the activation of human FLS in RA patients (20). *Yang* et al. found that RA T cells with diminished ROS production are spontaneously biased to differentiate into IFNγ and IL-17-producing proinflammatory T cells, which play a central role for disease progression (61). Thus, strategies that upregulate ROS levels in RA T cells and rebalance the ROS signaling systems might be promising for therapeutic purposes. This discovery strengthens the assumption that PL might be a promising agent for treating autoimmune diseases such as RA, SLE or EAE. However, one hindrance in the application of PL is its low water solubility. Therefore, water soluble derivates of PL that possess similar biological functions are currently under investigation (62, 63).

Also, of high clinical importance may be our second finding: PL differentially affects malignant Jurkat T leukemia cells (increase of GSH and tumor cell killing) and primary T cells (decrease of GSH and immunosuppression). This may be explained by the fact that tumor cells usually contain a higher antioxidant capacity than untransformed cells (e.g., Trx1 and GSH systems). Thereby, they can keep their redox balance more stable. For example, the GSH synthesis in tumor cells can be increased significantly during oxidative stress by upregulating the cystine/glutamate antiporter SLC7A11 and glutamate cysteine ligase modifier subunit (GCLM). Aside from the biosynthesis, tumor cells can also regenerate GSH by upregulating the production of nicotinamide adenine dinucleotide phosphate (NADPH) (64). A functional immune response plays a decisive role for the outcome of cancer. Therefore, when discussing the efficacy of PL against cancer, potential T cell immunosuppression by PL should be considered. Given that PL has a prooxidative effect on T cells, and that T cells are more susceptible to ROS than tumor cells, it is likely that the immunosuppressive effects of PL are more pronounced than the cytotoxic effects on tumor cells. This may be particularly important if cancer patients receive T cell-based immunotherapies (e.g., CAR T cells or checkpoint inhibitors). The sensitivity of T cells to low concentrations of PL further aggravates this situation, as the publications on anticancer effects of PL often show significant inhibition only with concentrations of 5 μM PL or higher (2, 5). In the future, it should also be investigated whether PL has a differential impact on other immune cell types and whether this involves similar molecular mechanisms as observed in T cells.

In summary, we have shown for the first time that PL differentially influences the fate of human primary T cells. Moreover, we have uncovered a molecular mechanism by which PL controls human T cell activation: PL induces a prooxidative milieu in primary human T cells by increasing ROS and depleting GSH. Thereby, it inhibits costimulation-induced T cell activation and proliferation. Importantly, PL diminishes T_H_17 differentiation while it enhances T_reg_ differentiation potentially by inhibiting HIF-1α. Furthermore, in T cells from patients with different autoimmune diseases PL inhibits the expression of the T cell activation markers CD69 and CD25 as well as production of IFNγ and IL-2. Together, these data suggest that PL may provide a new therapeutic option for chronic T_H_17-related inflammatory diseases. Simultaneously, it should be considered that during immunotherapies of cancer (e.g., CAR T cells or checkpoint inhibitors), in which T cell-mediated anti-tumor responses play a decisive role for the outcome of the disease, a treatment with PL may be harmful.

## Data Availability Statement

All datasets generated for this study are included in the article/Supplementary Material.

## Ethics Statement

The studies involving human participants were reviewed and approved by Ethics Committee of the Heidelberg University. The patients/participants provided their written informed consent to participate in this study.

## Author Contributions

JL and YS: conceptualization. JL, JZ, BJ, EB, BN, and GW: methodology. JL, JZ, BJ, EB, CO, RB, and TR: investigation. YS and NB: resources. JL, JZ, and YS: drafting of the article. JL, EB, KH, and YS: critical revision of the article. YS: supervision and funding acquisition. All authors have approved the final version of the manuscript.

## Conflict of Interest

The authors declare that the research was conducted in the absence of any commercial or financial relationships that could be construed as a potential conflict of interest.
