# Supplementary material for: Piperlongumine Acts as an Immunosuppressant by Exerting Prooxidative Effects in Human T Cells Resulting in Diminished TH17 but Enhanced Treg Differentiation
Source: Front Immunol. 2020 Jun 12;11:1172. doi: 10.3389/fimmu.2020.01172 (PMC7303365; doi:10.3389/fimmu.2020.01172)
Supplement: Supplementary file 1 [file Data_Sheet_1.pdf]

Supplementary Material

Piperlongumine acts as an immunosuppressant by exerting prooxidative effects in human T cells resulting in diminished T_H17 but enhanced T_{reg} differentiation

Jie Liang[1,2], Jacqueline D. Ziegler[1], Beate Jahraus[1], Christian Orlik[1], Renata Blatnik[3], Norbert Blank[4], Beate Niesler[5, 6], Guido Wabnitz[1], Thomas Ruppert[3], Katrin Hübner[1], Emre Balta[1], and Yvonne Samstag[1*]

***Correspondence**: yvonne.samstag@urz.uni-heidelberg.de

1 Supplementary Data

LC-MS measurements

Nanoflow LC-MS analysis was performed with a NanoAcquity UPLC liquid chromatography (Waters, Eschborn, Germany) system coupled to an Orbitrap XL or with an Ultimate 3000 liquid chromatography system coupled to an Orbitrap Q Exactive mass spectrometer (Thermo-Fischer, Bremen, Germany). When analyzed on the NanoAcquity UPLC – Orbitrap XL mass spectrometer, samples were loaded to a NanoAcquity Symmetry C18 Trap column (particle size 5 µm, inner diameter 180 µm x 20 mm, Waters) at a flow of 15 µL/min for 5 min of 0.5 % solvent B. Peptides were separated using a NanoAcquity M-Class peptide BEH C18 analytical column (particle size 1.7 µm, inner diameter 75 µm x 250 mm, Waters) with a 30 min linear gradient (3-40 % B) with a flow rate of 300 nL/min. Solvent A was 0.1% formic acid (FA; ProteoChem, Denver, CO, USA) in H_2O (Biosolve) and solvent B was composed of 0.1% FA (ProteoChem), 10% H_2O (Biosolve) and 89.9 % ACN (Biosolve). The mass spectrometer was operated in data-dependent acquisition mode, automatically switching between MS and MS^2. MS spectra (m/z 400–1600) were acquired in the Orbitrap at 60,000 (m/z 400) resolution. Collision induced dissociation MS^2 spectra were generated for up to 10 precursors with normalized collision energy of 35 % in the ion trap. When analyzed on the Ultimate 3000 – Q Exactive mass spectrometer, samples were delivered to an in-house packed analytical column (inner diameter 75 µm x 20 cm; CS – Chromatographie Service GmbH, Langerwehe, Germany) filled with 1.9 µm ReprosilPur-AQ 120 C18 material (Dr. Maisch, Ammerbuch-Entringen, Germany). Solvent A was 0.1 % formic acid (FA; ProteoChem, Denver, CO, USA) in H_2O (Biosolve) and solvent B was composed of 0.1 % FA (ProteoChem), 10 % H_2O (Biosolve) and 89.9 % ACN (Biosolve). Sample was loaded to the analytical column for 20 min with 3 % B at 550 nL/min flow rate. Peptides were separated with a 30 min linear gradient (3-40 % B) with a flow rate of 300 nL/min. The mass spectrometer was operated in data-dependent acquisition mode, automatically switching between MS, acquired at 60,000 (m/z 400) resolution, and MS^2 spectra, generated for up to 15 precursors with normalized collision energy of 27 % in the HCD cell and measured in the Orbitrap at 15,000 resolution.

2 Supplementary Figures

2.1 Supplementary Figure 1

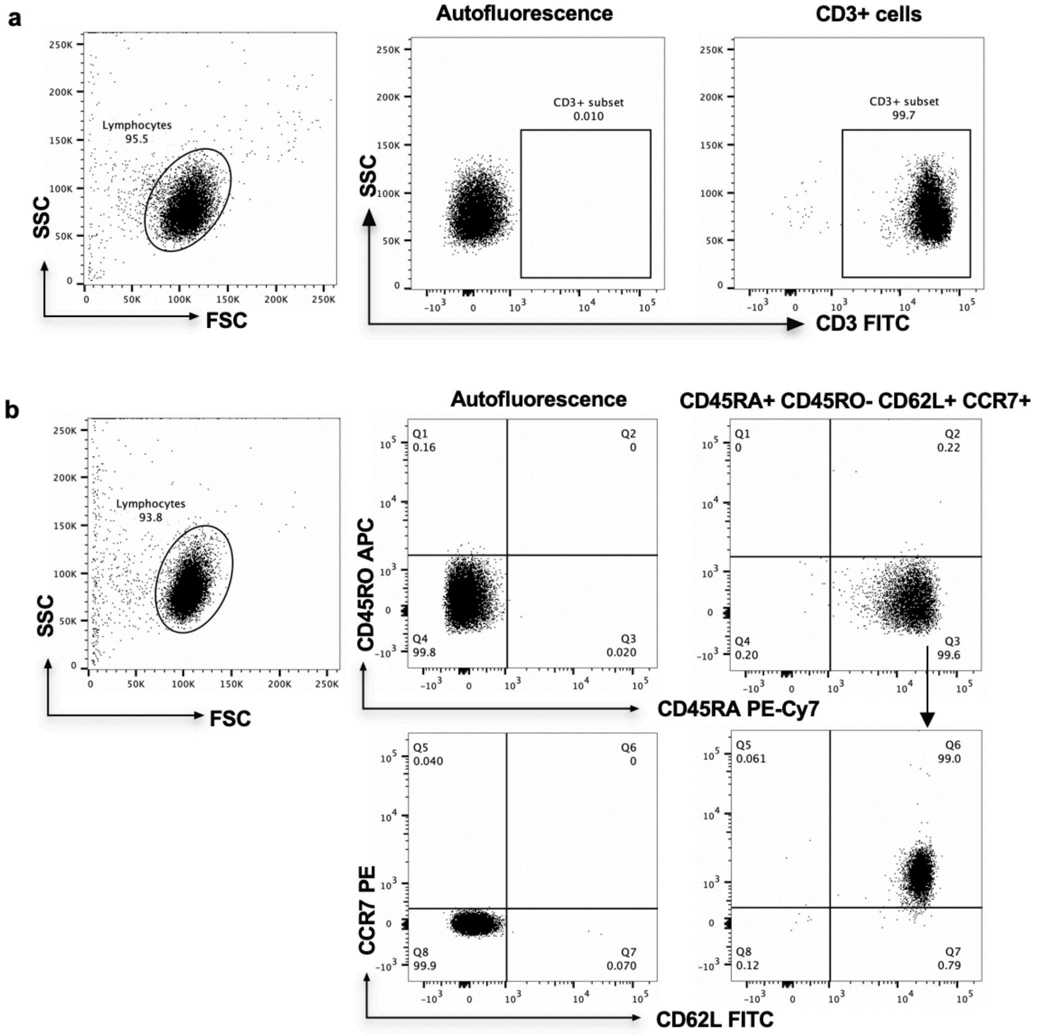

Supplementary Figure 1. Purity of MACS-separated primary human pan-T cells and naïve CD4^{+} cells (a) Purified primary human pan-T cells were stained with CD3, and detected by the flow cytometry. **(b)** Purified primary human naïve CD4^{+} cells were stained with CD45RA, CD45RO, CCR7, CD62L, and analysed with flow cytometry. Representative flow cytometry plots show the gating strategy.

2.2 Supplementary Figure 2

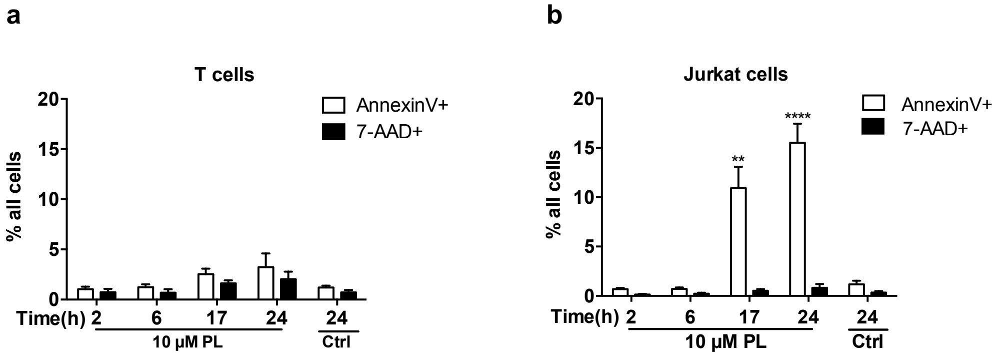

Supplementary Figure 2. PL is not cytotoxic to primary human T cells but to Jurkat cells (a) Primary human T cells or **(b)** Jurkat cells were treated with DMSO (Ctrl) or 10 µM PL for the indicated time points and then stained with AnnexinV and 7-AAD. Signals were detected by flow cytometry. Shown are the quantification of early apoptotic (AnnexinV+) or dead (7-AAD+) cells from the whole cell population ($n = 3$; mean; SEM; $^{**}p < 0.01$, $^{****}p < 0.0001$).

2.3 Supplementary Figure 3

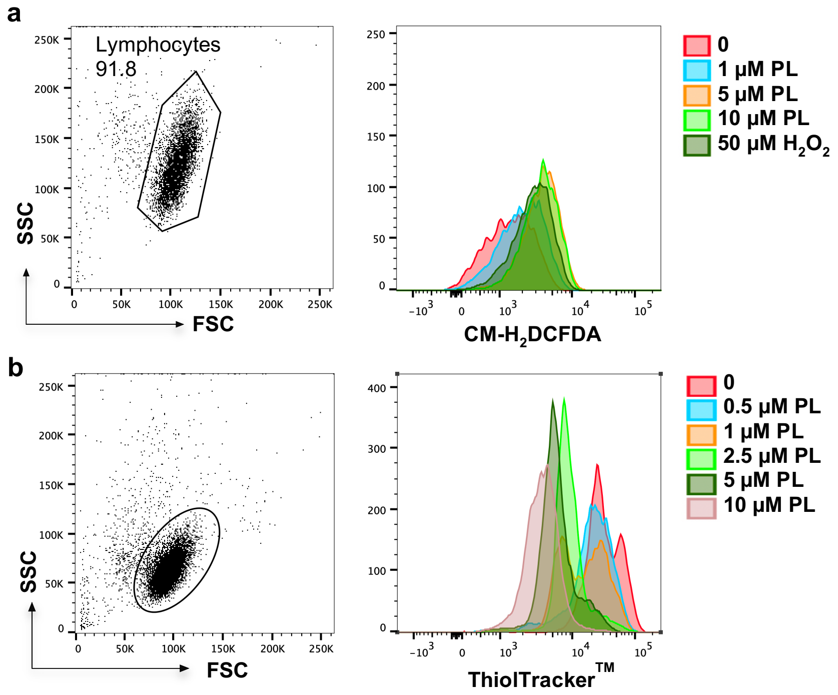

Supplementary Figure 3. Flow cytometry analysis of intracellular ROS and GSH in T cell. Representative dot plots showing gating on viable T cells (left panels) and histograms showing the MFI of the respective dyes (right panels) for **(a)** CM-H_2DCFDA (ROS) and **(b)** ThiolTracker™ (GSH) staining.

2.4 Supplementary Figure 4

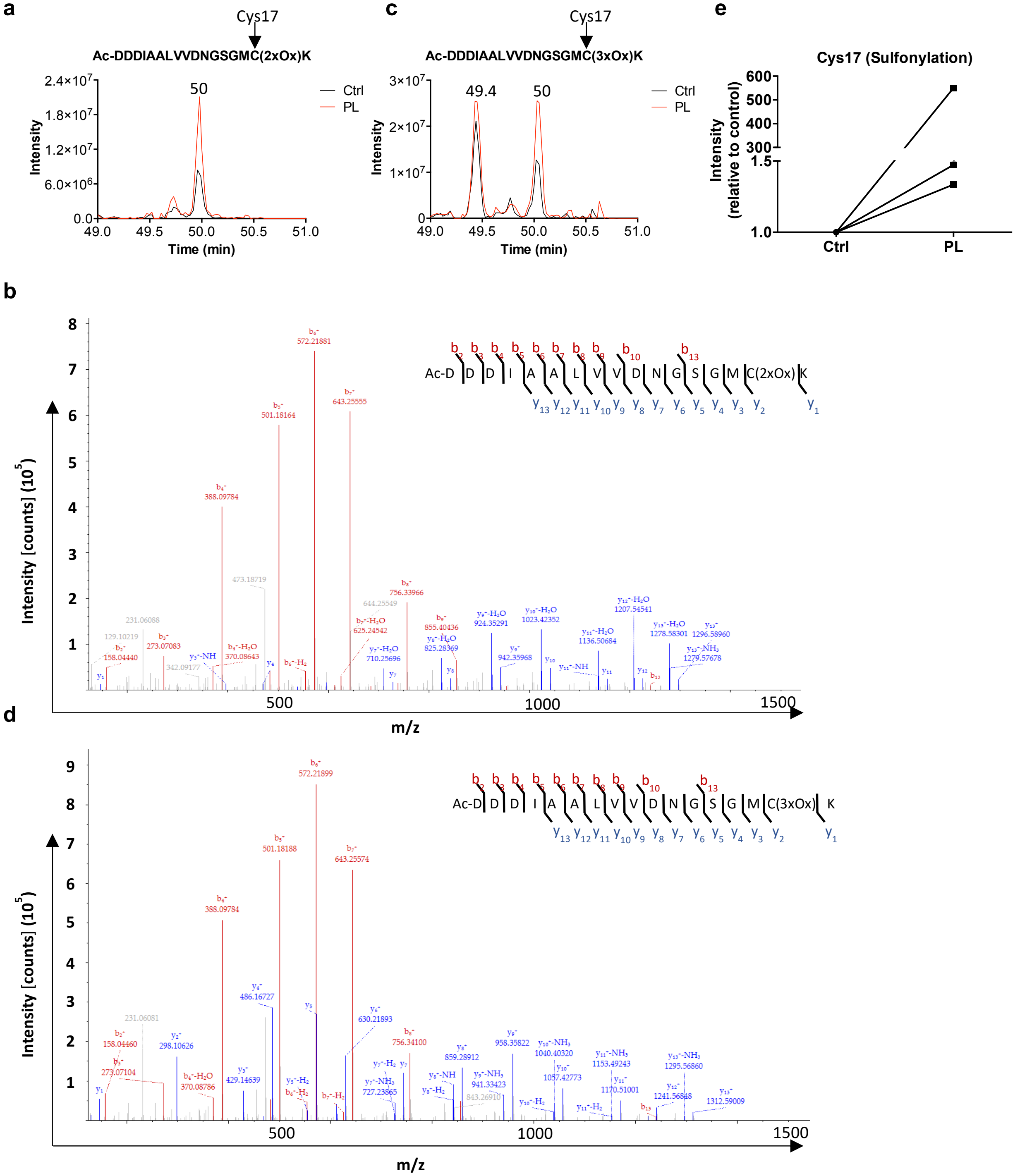

Supplementary Figure 4. PL oxidizes Cys17 in peptide Ac-DDDIAALVVDNGSGMCK of actin (a, c) Elution profile of peptide Ac-DDDIAALVVDNGSGMCK of actin in control and PL-treated samples with oxidation of Cys17 to **(a)** sulfinic (2xOx) or **(c)** sulfonic (3xOx) acid. Both peptides eluted at 50.0 min in control and PL-treated samples. Signal intensities increased for both peptides with PL-treatment. Chromatographic peak identities were confirmed with MS^2 spectra.

Peak areas calculated with the Skyline software were used for relative quantification of peptides. **(b, d)** MS^2 for the peptide Ac-DDDIAALVVDNGSGMCK of actin with oxidization of Cys17 to **(b)** sulfinic (2xOx) or **(d)** sulfonic (3xOx) acid. MS^2 spectra for both peptides were identified at 50.0 min. Both peptides were detected with cleaved N-terminal methionine and N-terminal acetylation on the first aspartic acid. Peptide sequence with vertical lines represents detected MS^2 ions and peptide sequence coverage. Red and blue peaks in MS^2 spectra represent b and y ions, respectively. **(e)** Sulfonylated (SO_3H) form of Cys17 in peptide Ac-DDDIAALVVDNGSGMCK of actin was higher upon PL treatment compared to the control in three biological replicates ($n = 3$). The intense signal at 49.4 min in (C) is a different peptide sharing the same precursor mass.

2.5 Supplementary Figure 5

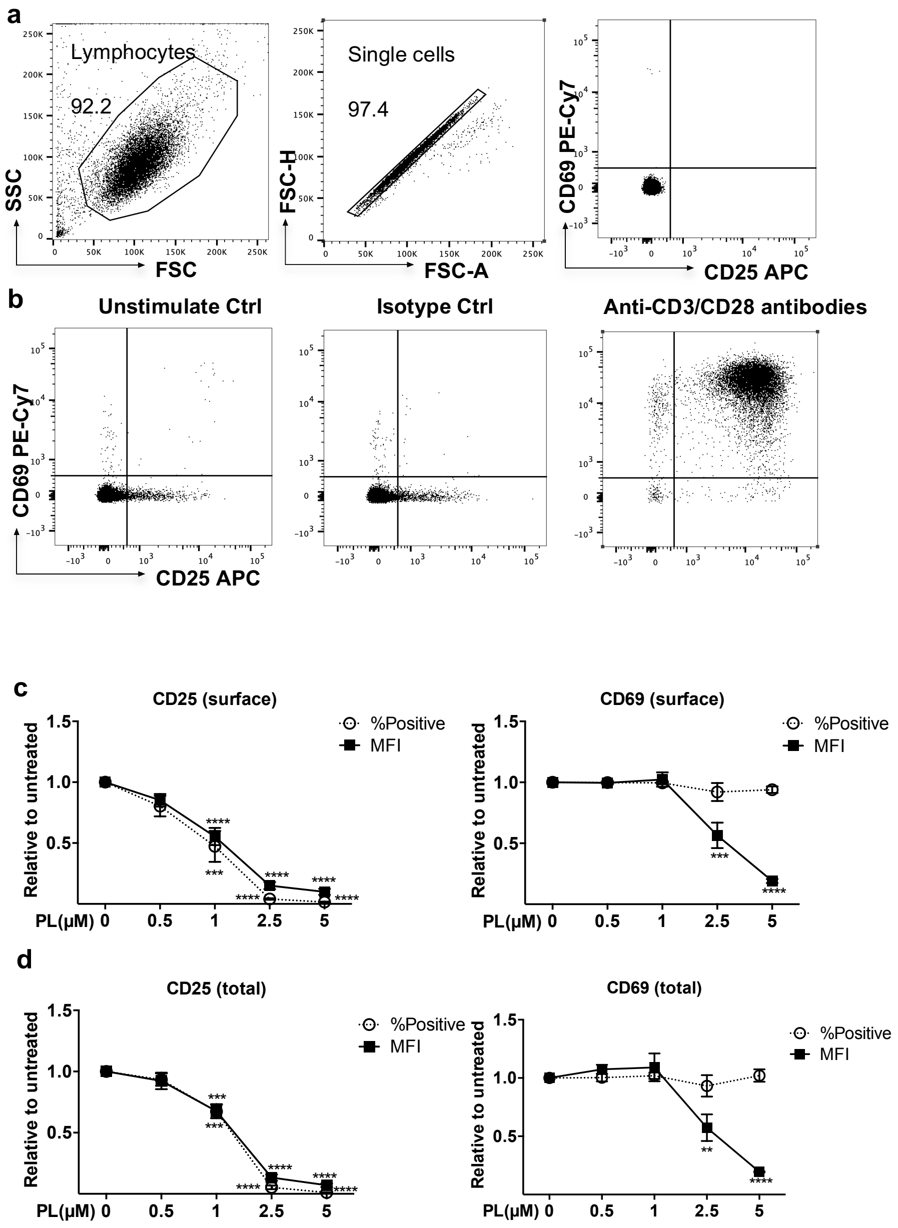

Supplementary Figure 5. PL inhibits the expression of costimulation-induced CD25 and CD69 in T cells (a) Representative flow cytometry dot plots show the gating strategy for CD25 and CD69 stained T cells. **(b)** Representative flow cytometry dot plots of CD25 and CD69 stained T cells, which are unstimulated, stimulated with anti-CD3/CD28 antibodies or isotype control antibodies. Flow cytometry analysis of CD25 (left) and CD69 (right) **(c)** surface and **(d)** total expression in T cells after stimulation with anti-CD3/CD28 antibodies for 24 h. Given are expression levels relative to control in terms of % positive cells and MFI (n = 3; mean; SEM; $^{**}p < 0.01$, $^{***}p < 0.001$, $^{****}p < 0.0001$).

2.6 Supplementary Figure 6

a

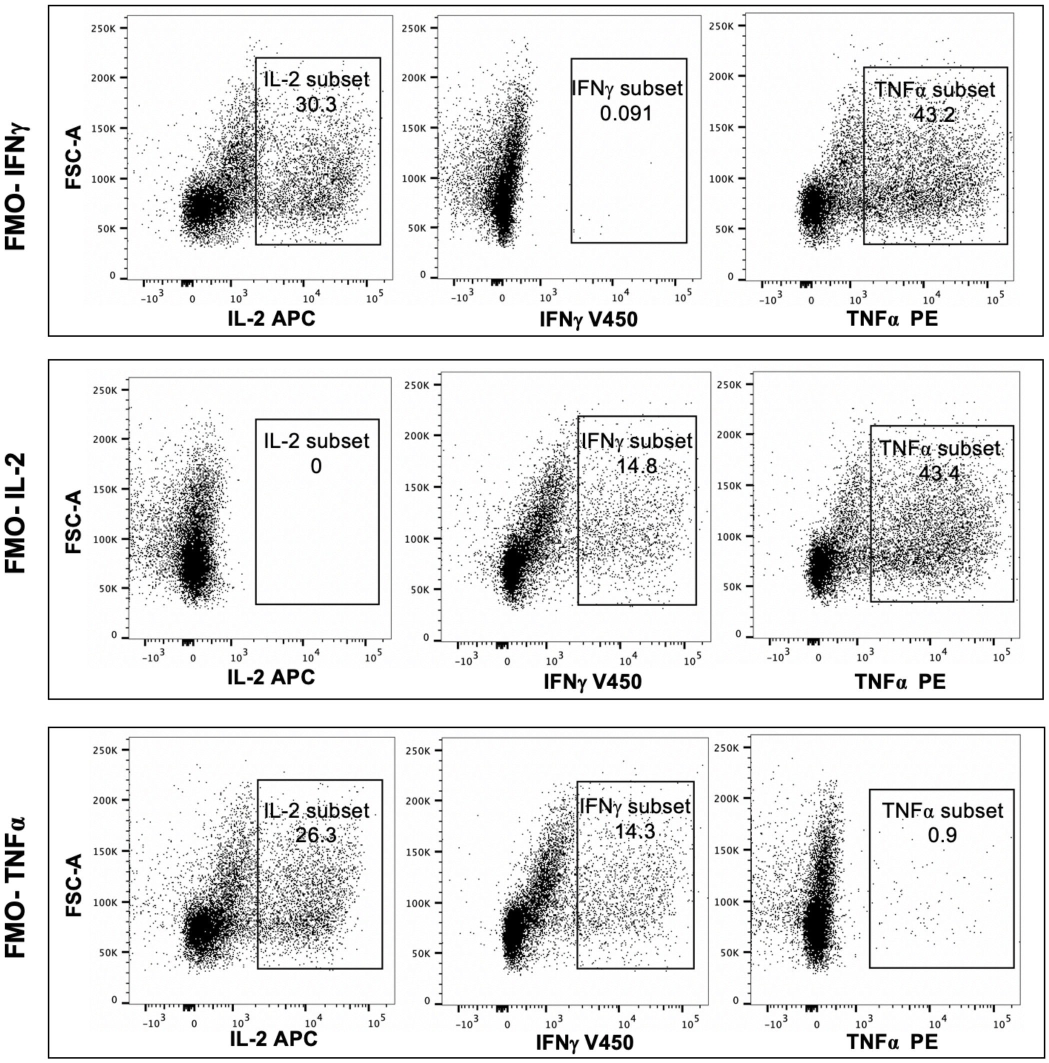

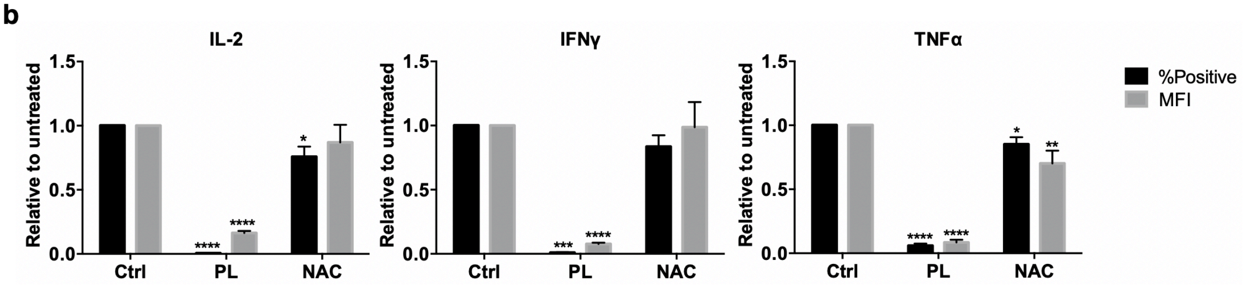

Supplementary Figure 6. Intracellular staining of IL-2, IFNγ and TNFα (a) Representative flow cytometry dot plots (Florescence Minus One (FMO)). **(b)** Quantification of IFNγ, TNFα and IL-2 detected by intracellular cytokine staining. Shown are ratios of 5 µM PL-treated or NAC-treated cells to the untreated control with regard to the % positive cells (black bars) and MFI (grey bars) ($n = 3$; mean; SEM; *$p < 0.05$, **$p < 0.01$, ***$p < 0.001$, ****$p < 0.0001$).

2.7 Supplementary Figure 7

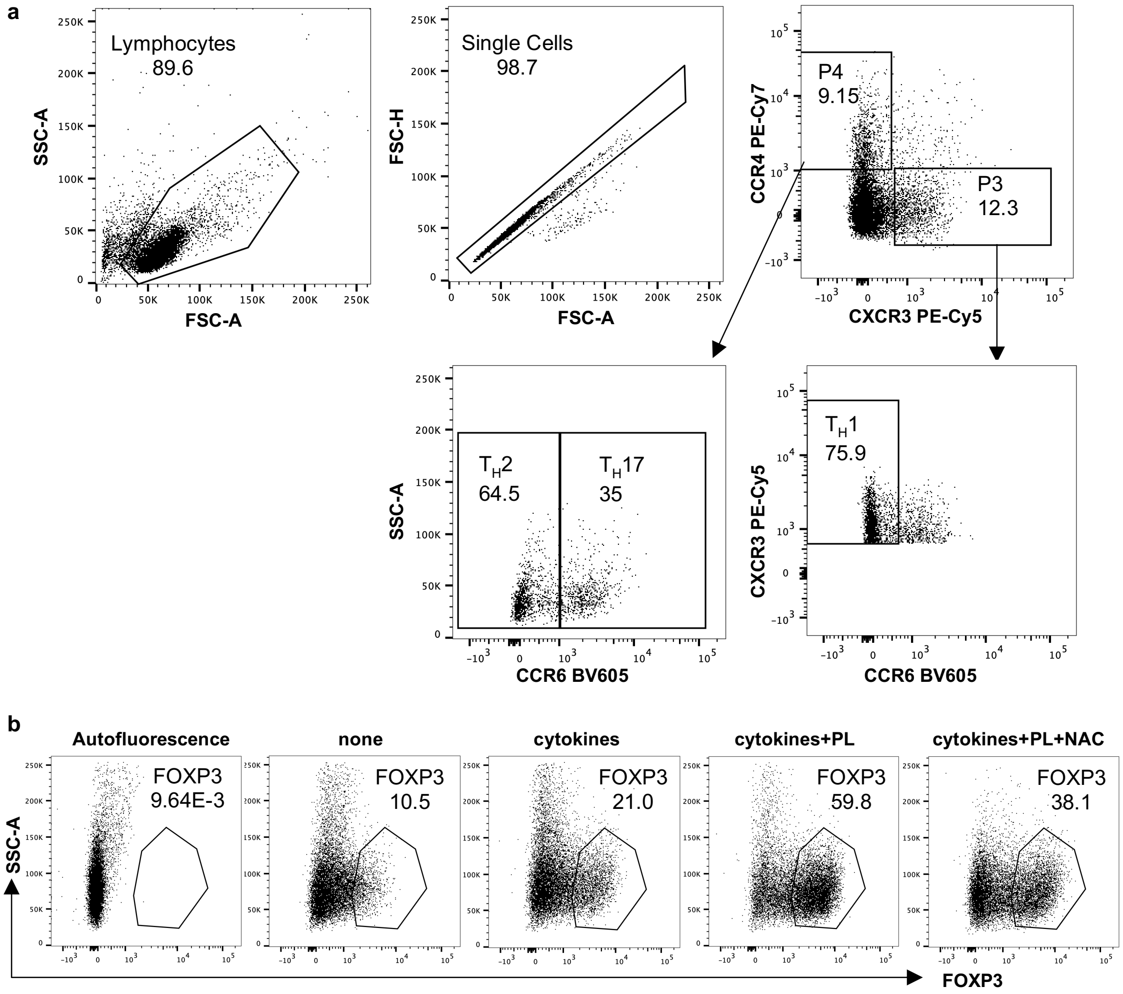

Supplementary Figure 7. Gating strategies for different T cell subtypes (a) The gating strategy for T_H1, T_H2 and T_H17 cells. **(b)** Representative flow cytometry dot plots of FOXP3-positive T cell populations.

2.8 Supplementary Figure 8

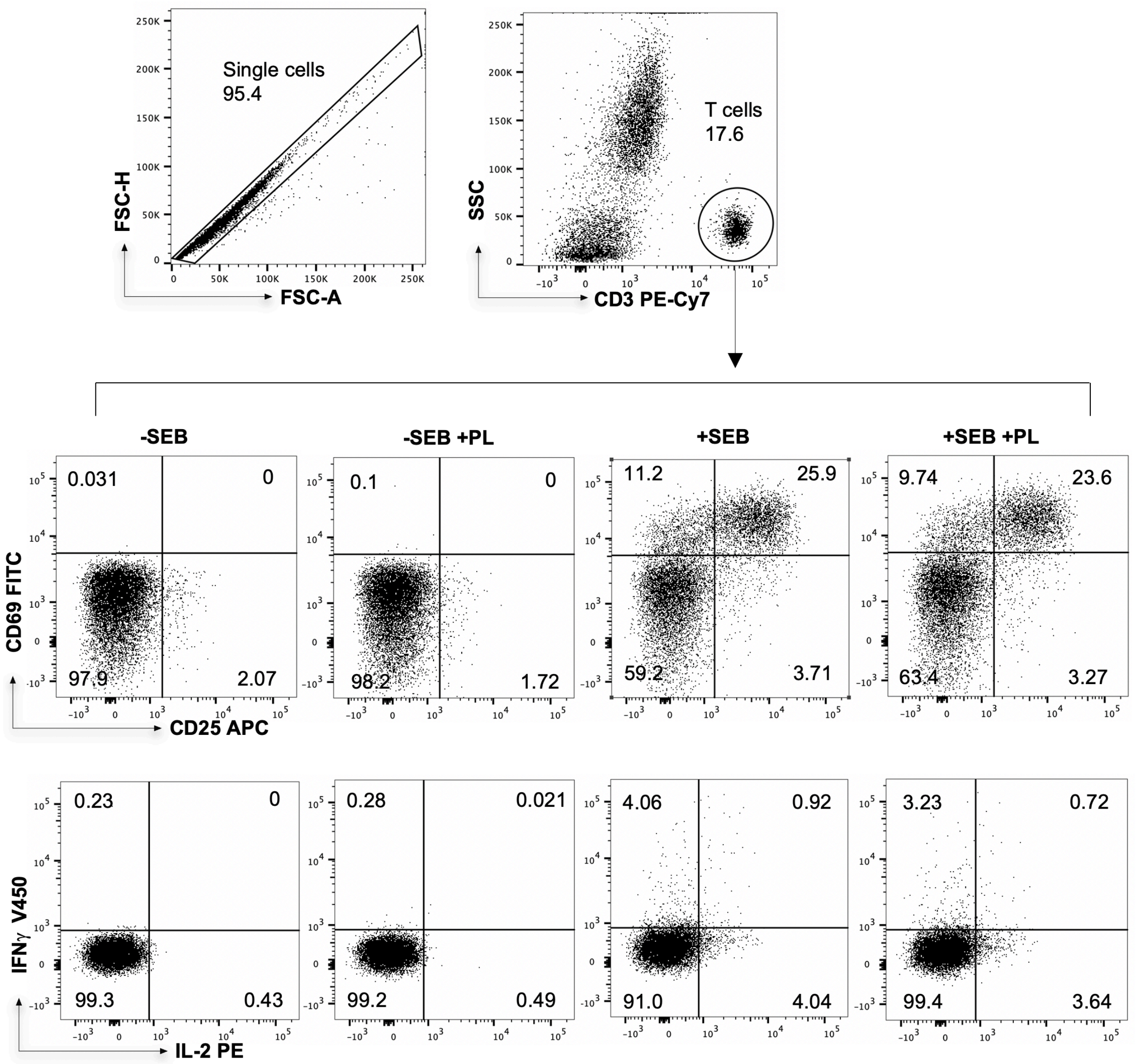

Supplementary Figure 8. Whole blood from patients. Gating strategy for T cells **(upper panel).** Representative flow cytometry dot plots showing expression of CD25 and CD69 **(middle panel)**, or IFNγ and IL-2 (**lower panel**) in T cells of patients with or without 20 h of 5 µM PL treatment.
